# Chemistry and biology of marine-derived *Trichoderma* metabolites

**DOI:** 10.1007/s13659-024-00433-3

**Published:** 2024-02-02

**Authors:** Yin-Ping Song, Nai-Yun Ji

**Affiliations:** Yantai Institute of Coastal Zone Research, Chinese Academy of Sciences, Yantai, 264003 People’s Republic of China

**Keywords:** *Trichoderma*, Metabolite, Terpene, Polyketide, Peptide, Bioactivity

## Abstract

Marine-derived fungi of the genus *Trichoderma* have been surveyed for pharmaceuticals and agrochemicals since 1993, with various new secondary metabolites being characterized from the strains of marine animal, plant, sediment, and water origin. Chemical structures and biological activities of these metabolites are comprehensively reviewed herein up to the end of 2022 (covering 30 years). More than 70 strains that belong to at least 18 known *Trichoderma* species have been chemically investigated during this period. As a result, 445 new metabolites, including terpenes, steroids, polyketides, peptides, alkaloids, and others, have been identified, with over a half possessing antimicroalgal, zooplankton-toxic, antibacterial, antifungal, cytotoxic, anti-inflammatory, and other activities. The research is highlighted by the molecular diversity and antimicroalgal potency of terpenes and steroids. In addition, metabolic relevance along with co-culture induction in the production of new compounds is also concluded. *Trichoderma* strains of marine origin can transform and degrade heterogeneous molecules, but these functions need further exploration.

**Graphical Abstract:**

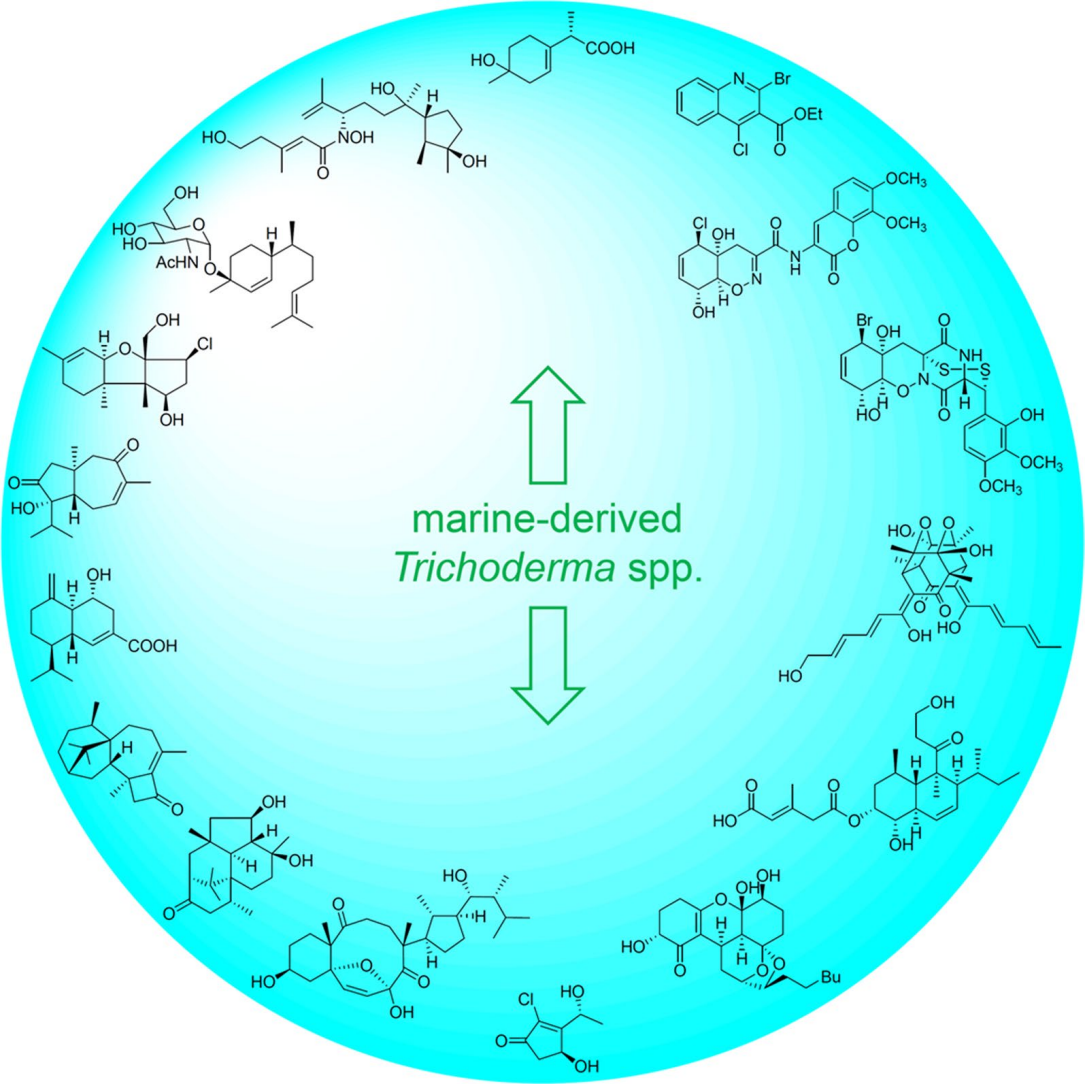

## Introduction

*Trichoderma* was first described as a genus of filamentous fungi by Persoon in 1794 [1]. It belongs to the family Hypocreaceae of the order Hypocreales in the class Sordariomycetes of the phylum Ascomycota, rather than the previous deuteromycetous taxa, according to the Dictionary of the Fungi (10th edition) [2]. Fungal species of this genus feature numerous conidia in varying shades of green, rapid growth rate, and high adaptability to various terrestrial and aquatic ecosystems. They are free-living or occur as mycoparasites and opportunistic, avirulent plant symbionts [3]. Their identification is difficult due to the few morphological characteristics available and the slight variations among different species [4]. Most of them can be linked to the teleomorphic *Hypocrea* species (Ascomycota), but this generic name has not been suggested to be further used by the proposal from Rossman et al. [5]. Several species were once assigned to other genera, such as *Aphysiostroma*, *Eidamia*, *Cordyceps*, *Gliocladium*, *Podostroma*, *Sarawakus*, *Sphaeria*, *Sporotrichum*, *Stilbella*, *Stilbum*, and *Thuemenella*, and 65 names inclusive of the basionyms of accepted species in *Trichoderma* and other genera are not currently adopted [6]. Totally, 254 species and two varieties with at least 1100 strains were characterized until 2015 [6, 7], and new species have been continually recognized thereafter [8–11].

Since the initial work by Weindling regarding the parasitism of *T.* *lignorum* on other fungi in the early 1930s [12, 13], *Trichoderma* species have achieved great successes to suppress plant diseases and promote plant growth in agriculture [7], and more than 250 *Trichoderma*-based products have been commercialized so far [14]. Their interactions with pathogens and plants appear complicated, but the production of bioactive secondary metabolites is regarded as one of the key mechanisms [15–17]. Investigations toward the antagonism of *Trichoderma* species against phytopathogenic fungi have resulted in the isolation and identification of a number of antifungal antibiotics, including terpenes, polyketides, peptides, and alkaloids [18]. A series of metabolites have been reported as plant growth promoters or inhibitors [19], and a plethora of metabolites with other activities, such as antibacterial, cytotoxic, and enzyme-inhibitory properties, have also been discovered [19–23]. Over the past eight decades, some a thousand new polar and nonpolar metabolites have been isolated and identified from *Trichoderma* species of various origin including terrestrial and marine environments.

**Fig. 1:**
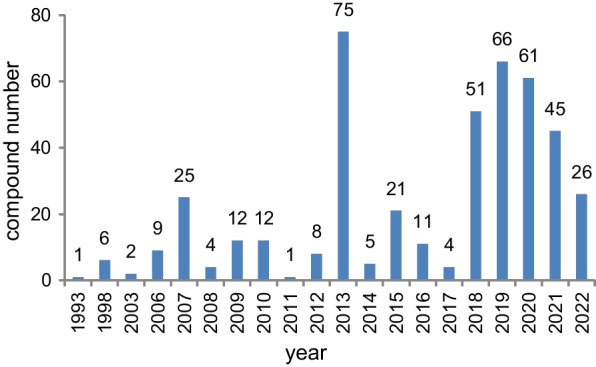
Numbers of new compounds from marine-derived *Trichoderma* during 1993–2022

**Fig. 2:**
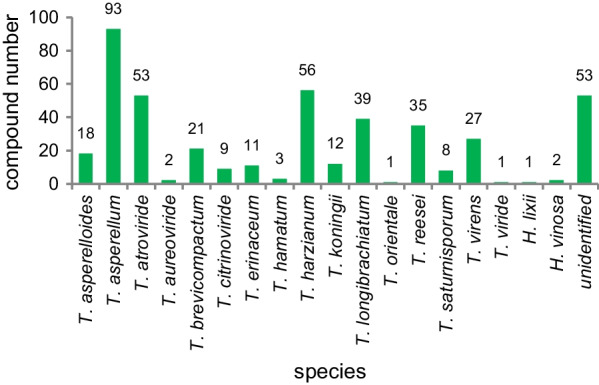
Numbers of new compounds from different marine-derived *Trichoderma* species

**Fig. 3:**
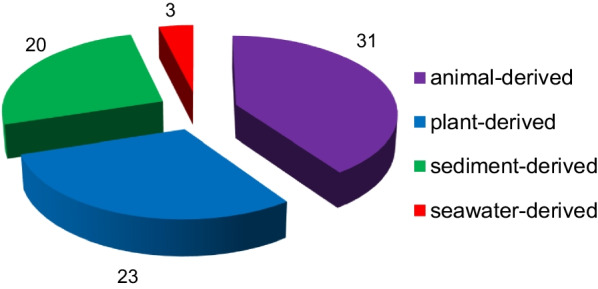
Sources and numbers of marine-derived *Trichoderma* strains possessing new compounds

**Fig. 4:**
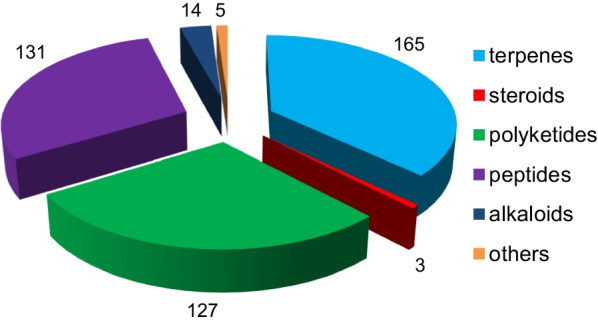
Types and numbers of new compounds from marine-derived *Trichoderma*

**Fig. 5:**
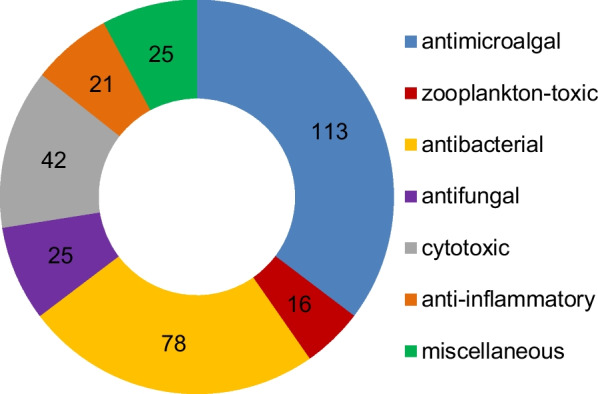
Numbers of bioactive new compounds from marine-derived *Trichoderma*

Since 1993, secondary metabolites from marine-derived *Trichoderma* have gradually been surveyed [24]. Until the end of 2022, 445 new compounds have been identified from 77 strains of *Trichoderma* fungi, 60 of which belong to 18 known species involving *T.* *asperelloides* (1 strain), *T.* *asperellum* (5), *T.* *atroviride* (9), *T.* *aureoviride* (1), *T.* *brevicompactum* (2), *T.* *citrinoviride* (4), *T.* *erinaceum* (1), *T.* *hamatum* (1), *T.* *harzianum* (12), *T.* *koningii* (3), *T.* *longibrachiatum* (8), *T.* *orientale* (1), *T.* *reesei* (4), *T.* *saturnisporum* (1), *T.* *virens* (4), *T.* *viride* (1), *H.* *lixii* (1), and *H.* *vinosa* (1) (Figs. 1, 2). These 77 *Trichoderma* strains have been isolated from animal (sponge (19 strains), mussel (4), fish (1), tunicate (2), sea star (2), sea fan (1), and soft coral (2)), plant (alga (18), mangrove (4), and halophyte (1)), sediment (20), and seawater (3) samples (Fig. 3). The new compounds (Tables 1, 2, 3) can be divided into terpenes, steroids, polyketides, peptides, alkaloids, and others (Fig. 4), of which 235 members possess antimicroalgal, zooplankton-toxic, antibacterial, antifungal, cytotoxic, anti-inflammatory, antiviral, phytotoxic, insect-toxic, zebrafish-toxic, antifouling, antioxidant, enzyme-inhibitory, NF-κB-inhibitory, anti-pulmonary fibrosis, anti-Aβ fibrillization, and neuroprotective activities (Fig. 5) [24–130]. The present review attempts to give a comprehensive compilation of these molecules and highlights their chemical diversity and biological activity.

**Table 1:** Terpenes (**1**–**165**) and steroids (**166**–**168**) from the marine-derived *Trichoderma*

No	Name	Bioactivity	Producer	Source	Locality	References
1	(7*S*)-1-Hydroxy-3-*p*-menthen-9-oic acid		*T.* *asperellum* cf44-2	Alga *Sargassum* sp.	Zhoushan Islands, China	[25]
2	(7*R*)-1-Hydroxy-3-*p*-menthen-9-oic acid		*T.* *asperellum* cf44-2	Alga *Sargassum* sp.	Zhoushan Islands, China	[25]
3	9-Cycloneren-3,7,11-triol	Zooplankton-toxic	*T.* *asperellum* cf44-2	Alga *Sargassum* sp.	Zhoushan Islands, China	[26]
4	11-Cycloneren-3,7,10-triol	Zooplankton-toxic	*T.* *asperellum* cf44-2	Alga *Sargassum* sp.	Zhoushan Islands, China	[26]
5	7,10-Epoxycycloneran-3,11,12-triol	Antibacterial and zooplankton-toxic	*T.* *asperellum* cf44-2	Alga *Sargassum* sp.	Zhoushan Islands, China	[26]
6	Cyclonerpyranol	Antimicroalgal	*T.* *asperellum* A-YMD-9-2	Alga *Gracilaria* *verrucosa*	Yangma Island, Yantai, China	[27]
7	3,7,11-Trihydroxycycloneran-10-one	Antimicroalgal	*T.* *asperellum* A-YMD-9-2	Alga *Gracilaria* *verrucosa*	Yangma Island, Yantai, China	[28]
8	Cycloneran-3,7,10,11-tetraol	Antimicroalgal	*T.* *asperellum* A-YMD-9-2	Alga *Gracilaria* *verrucosa*	Yangma Island, Yantai, China	[28]
9	Cycloneran-3,7,11-triol	Antimicroalgal	*T.* *asperellum* A-YMD-9-2	Alga *Gracilaria* *verrucosa*	Yangma Island, Yantai, China	[28]
10	7,10*S*-Epoxycycloneran-3,15-diol	Antimicroalgal	*T.* *asperellum* A-YMD-9-2	Alga *Gracilaria* *verrucosa*	Yangma Island, Yantai, China	[28]
11	7,10*R*-Epoxycycloneran-3,15-diol	Antimicroalgal	*T.* *asperellum* A-YMD-9-2	Alga *Gracilaria* *verrucosa*	Yangma Island, Yantai, China	[28]
12	(10*Z*)-15-Acetoxy-10-cycloneren-3,7-diol	Antimicroalgal	*T.* *asperellum* A-YMD-9-2	Alga *Gracilaria* *verrucosa*	Yangma Island, Yantai, China	[28]
13	(10*E*)-12-Acetoxy-10-cycloneren-3,7-diol		*T.* *harzianum* P1-4	Sediment	Bohai Sea, China	[29]
14	12-Acetoxycycloneran-3,7-diol		*T.* *harzianum* P1-4	Sediment	Bohai Sea, China	[29]
15	11-Methoxy-9-cycloneren-3,7-diol	Antimicroalgal and antibacterial	*T.* *harzianum* X-5	Alga *Laminaria* *japonica*	Chang Islands, China	[30]
16	10-Cycloneren-3,5,7-triol	Antimicroalgal and antibacterial	*T.* *harzianum* X-5	Alga *Laminaria* *japonica*	Chang Islands, China	[30]
17	Methyl 3,7-dihydroxy-15-cycloneranate	Antimicroalgal and antibacterial	*T.* *harzianum* X-5	Alga *Laminaria* *japonica*	Chang Islands, China	[30]
18	5-Hydroxyepicyclonerodiol oxide	Antimicroalgal and antibacterial	*T.* *hamatum* Z36-7	Alga *Grateloupia* sp.	Zhoushan Islands, China	[31]
19	4-Hydroxyepicyclonerodiol oxide	Antimicroalgal and antibacterial	*T.* *hamatum* Z36-7	Alga *Grateloupia* sp.	Zhoushan Islands, China	[31]
20	Cyclonerodiol A		*T.* *erinaceum* F1-1	Sea star *Acanthaster* *planci*	Hainan Sanya National Coral Reef Reserve, China	[32]
21	Cyclonerodiol B		*T.* *erinaceum* F1-1	Sea star *Acanthaster* *planci*	Hainan Sanya National Coral Reef Reserve, China	[32]
22	(10*E*)-Isocyclonerotriol	Antimicroalgal and antibacterial	*T.* *citrinoviride* A-WH-20-3	Alga *Laurencia* *okamurai*	Weihai, China	[33]
23	(10*Z*)-Isocyclonerotriol	Antimicroalgal and antibacterial	*T.* *citrinoviride* A-WH-20-3	Alga *Laurencia* *okamurai*	Weihai, China	[33]
24	Isoepicyclonerodiol oxide	Antimicroalgal and antibacterial	*T.* *asperelloides* RR-dl-6-11	Alga *Rhodomela* *confervoides*	Dalian, China	[34]
25	Cycloner-3-en-7,11-diol	Antimicroalgal	*T.* *asperelloides* RR-dl-6-11	Alga *Rhodomela* *confervoides*	Dalian, China	[34]
26	11,12,15-Trinorcycloneran-3,7,10-triol	Antimicroalgal	*T.* *asperellum* A-YMD-9-2	Alga *Gracilaria* *verrucosa*	Yangma Island, Yantai, China	[28]
27	Norepicyclonerodiol oxide	Antimicroalgal and antibacterial	*T.* *asperelloides* RR-dl-6-11	Alga *Rhodomela* *confervoides*	Dalian, China	[34]
28	Cyclonerin A	Antimicroalgal	*T.* *asperellum* A-YMD-9-2	Alga *Gracilaria* *verrucosa*	Yangma Island, Yantai, China	[27]
29	Cyclonerin B	Antimicroalgal	*T.* *asperellum* A-YMD-9-2	Alga *Gracilaria* *verrucosa*	Yangma Island, Yantai, China	[27]
30	Deoxycyclonerin A	Antimicroalgal	*T.* *asperellum* A-YMD-9-2	Alga *Gracilaria* *verrucosa*	Yangma Island, Yantai, China	[27]
31	Deoxycyclonerin B	Antimicroalgal	*T.* *asperellum* A-YMD-9-2	Alga *Gracilaria* *verrucosa*	Yangma Island, Yantai, China	[27]
32	Deoxycyclonerin C	Antimicroalgal	*T.* *asperellum* A-YMD-9-2	alga *Gracilaria* *verrucosa*	Yangma Island, Yantai, China	[27]
33	Deoxycyclonerin D	Antimicroalgal	*T.* *asperellum* A-YMD-9-2	Alga *Gracilaria* *verrucosa*	Yangma Island, Yantai, China	[27]
34	Cyclonerinal	Antimicroalgal	*T.* *asperellum* A-YMD-9-2	Alga *Gracilaria* *verrucosa*	Yangma Island, Yantai, China	[27]
35	Cyclonerizole	Antimicroalgal	*T.* *asperellum* A-YMD-9-2	Alga *Gracilaria* *verrucosa*	Yangma Island, Yantai, China	[27]
36	5′-Acetoxy-deoxycyclonerin B	Antimicroalgal	*T.* *asperellum* A-YMD-9-2	Alga *Gracilaria* *verrucosa*	Yangma Island, Yantai, China	[35]
37	5′-Acetoxy-deoxycyclonerin D	Antimicroalgal	*T.* *asperellum* A-YMD-9-2	Alga *Gracilaria* *verrucosa*	Yangma Island, Yantai, China	[35]
38	Trichaspside A	Antibacterial and zooplankton-toxic	*T.* *asperellum* cf44-2	Alga *Sargassum* sp.	Zhoushan Islands, China	[26]
39	Bisabolan-1,10,11-triol	Antimicroalgal and antibacterial	*T.* *asperellum* cf44-2	Alga *Sargassum* sp.	Zhoushan Islands, China	[25, 34]
40	Trichobisabolin A	Antimicroalgal	*T.* *asperellum* Y6-2	Alga *Chondrus* *ocellatus*	Yangma Island, Yantai, China	[36]
41	Trichobisabolin B	Antimicroalgal	*T.* *asperellum* Y6-2	Alga *Chondrus* *ocellatus*	Yangma Island, Yantai, China	[36]
42	Trichobisabolin C	Antimicroalgal	*T.* *asperellum* Y6-2	Alga *Chondrus* *ocellatus*	Yangma Island, Yantai, China	[36]
43/44	Trichobisabolins O_1_/O_2_	Antimicroalgal	*T.* *brevicompactum* A-DL-9-2	Alga *Chondria* *tenuissima*	Dalian, China	[37]
45	Trichobisabolin P	Antimicroalgal	*T.* *brevicompactum* A-DL-9-2	Alga *Chondria* *tenuissima*	Dalian, China	[37]
46	Trichobisabolin T	Antimicroalgal and antibacterial	*T.* *asperelloides* RR-dl-6-11	Alga *Rhodomela* *confervoides*	Dalian, China	[34]
47	Trichobisabolin U	Antimicroalgal and antibacterial	*T.* *asperelloides* RR-dl-6-11	Alga *Rhodomela* *confervoides*	Dalian, China	[34]
48	Trichobisabolin V	Antimicroalgal and antibacterial	*T.* *asperelloides* RR-dl-6-11	Alga *Rhodomela* *confervoides*	Dalian, China	[34]
49	Trichobisabolin W	Antimicroalgal and antibacterial	*T.* *asperelloides* RR-dl-6-11	Alga *Rhodomela* *confervoides*	Dalian, China	[34]
50	Trichobisabolin X	Antimicroalgal	*T.* *asperelloides* RR-dl-6-11	Alga *Rhodomela* *confervoides*	Dalian, China	[34]
51	Trichodermaerin A		*T.* *erinaceum* F1-1	Sea star *Acanthaster* *planci*	Hainan Sanya National Coral Reef Reserve, China	[32]
52	12-Nor-11-acetoxybisabolen-3,6,7-triol	Antimicroalgal and antibacterial	*T.* *asperellum* cf44-2	Alga *Sargassum* sp.	Zhoushan Islands, China	[25]
53	Trichaspside B	Antibacterial and zooplankton-toxic	*T.* *asperellum* cf44-2	Alga *Sargassum* sp.	Zhoushan Islands, China	[26]
54	Trichaspside C	Antimicroalgal and antibacterial	*T.* *asperellum* A-YMD-9-2	Alga *Gracilaria* *verrucosa*	Yangma Island, Yantai, China	[38]
55	Trichaspside D	Antimicroalgal and antibacterial	*T.* *asperellum* A-YMD-9-2	Alga *Gracilaria* *verrucosa*	Yangma Island, Yantai, China	[38]
56	Trichaspside E	Antimicroalgal and antibacterial	*T.* *asperellum* A-YMD-9-2	Alga *Gracilaria* *verrucosa*	Yangma Island, Yantai, China	[38]
57	Trichobisabolin D	Antimicroalgal and zooplankton-toxic	*T.* *asperellum* Y6-2	Alga *Chondrus* *ocellatus*	Yangma Island, Yantai, China	[36]
58	Trichobisabolin E	Antimicroalgal	*T.* *asperellum* Y6-2	Alga *Chondrus* *ocellatus*	Yangma Island, Yantai, China	[36]
59	Trichobisabolin F	Antimicroalgal	*T.* *asperellum* Y6-2	Alga *Chondrus* *ocellatus*	Yangma Island, Yantai, China	[36]
60	Trichobisabolin G	Antimicroalgal	*T.* *asperellum* Y6-2	Alga *Chondrus* *ocellatus*	Yangma Island, Yantai, China	[36]
61	Trichobisabolin H	Antimicroalgal and zooplankton-toxic	*T.* *asperellum* Y6-2	Alga *Chondrus* *ocellatus*	Yangma Island, Yantai, China	[36]
62	Trichobisabolin I	Antimicroalgal and antibacterial	*T.* *asperellum* A-YMD-9-2	Alga *Gracilaria* *verrucosa*	Yangma Island, Yantai, China	[38]
63	Trichobisabolin J	Antimicroalgal and antibacterial	*T.* *asperellum* A-YMD-9-2	Alga *Gracilaria* *verrucosa*	Yangma Island, Yantai, China	[38]
64	Trichobisabolin K	Antimicroalgal and antibacterial	*T.* *asperellum* A-YMD-9-2	Alga *Gracilaria* *verrucosa*	Yangma Island, Yantai, China	[38]
65	Trichobisabolin L	Antimicroalgal and antibacterial	*T.* *asperellum* A-YMD-9-2	Alga *Gracilaria* *verrucosa*	Yangma Island, Yantai, China	[38]
66	Trichobisabolin M	Antimicroalgal and antibacterial	*T.* *atroviride* RR-dl-3-9	Alga *Rhodomela* *confervoides*	Dalian, China	[39]
67	Trichobisabolin N	Antimicroalgal and antibacterial	*T.* *atroviride* RR-dl-3-9	Alga *Rhodomela* *confervoides*	Dalian, China	[39]
68	Trichobisabolin Q	Antimicroalgal and antibacterial	*T.* *asperelloides* RR-dl-6-11	Alga *Rhodomela* *confervoides*	Dalian, China	[34]
69	Trichobisabolin R	Antimicroalgal and antibacterial	*T.* *asperelloides* RR-dl-6-11	Alga *Rhodomela* *confervoides*	Dalian, China	[34]
70	Trichobisabolin S	Antimicroalgal and antibacterial	*T.* *asperelloides* RR-dl-6-11	Alga *Rhodomela* *confervoides*	Dalian, China	[34]
71	Trichobisabolin Y	Antimicroalgal and antibacterial	*T.* *asperelloides* RR-dl-6-11	Alga *Rhodomela* *confervoides*	Dalian, China	[34]
72	Trichobisabolin Z	Antimicroalgal and antibacterial	*T.* *asperelloides* RR-dl-6-11	Alga *Rhodomela* *confervoides*	Dalian, China	[34]
73		Anti-inflammatory	*T.* *brevicompactum* NTU439	Alga *Mastophora* *rosea*	Yilan coast, China	[40]
74		Anti-inflammatory	*T.* *brevicompactum* NTU439	Alga *Mastophora* *rosea*	Yilan coast, China	[40]
75		Anti-inflammatory	*T.* *brevicompactum* NTU439	Alga *Mastophora* *rosea*	Yilan coast, China	[40]
76		Anti-inflammatory	*T.* *brevicompactum* NTU439	Alga *Mastophora* *rosea*	Yilan coast, China	[40]
77	Trichobreol A	Antifungal	*Trichoderma* sp. TPU199 (cf. *T.* *brevicompactum*)	Unidentified alga	Palau	[41]
78	Trichobreol B	Antifungal	*Trichoderma* sp. TPU199 (cf. *T.* *brevicompactum*)	Unidentified alga	Palau	[41]
79	Trichobreol C	Antifungal	*Trichoderma* sp. TPU199 (cf. *T.* *brevicompactum*)	Unidentified alga	Palau	[41]
80	Trichobreol D	Antifungal	*Trichoderma* sp. TPU199 (cf. *T.* *brevicompactum*)	Unidentified alga	Palau	[42]
81	Trichobreol E	Antifungal	*Trichoderma* sp. TPU199 (cf. *T.* *brevicompactum*)	Unidentified alga	Palau	[42]
82	Trichodermol chlorohydrin	Antimicroalgal and antibacterial	*T.* *hamatum* Z36-7	Alga *Grateloupia* sp.	Zhoushan Islands, China	[31]
83	Trichodermarin G	Antimicroalgal and antifungal	*T.* *brevicompactum* A-DL-9-2	Alga *Chondria* *tenuissima*	Dalian, China	[43]
84	Trichodermarin H	Antimicroalgal and antifungal	*T.* *brevicompactum* A-DL-9-2	Alga *Chondria* *tenuissima*	Dalian, China	[43]
85	Trichodermarin I	Antimicroalgal and antifungal	*T.* *brevicompactum* A-DL-9-2	Alga *Chondria* *tenuissima*	Dalian, China	[43]
86	Trichodermarin J	Antimicroalgal	*T.* *brevicompactum* A-DL-9-2	Alga *Chondria* *tenuissima*	Dalian, China	[43]
87	Trichodermarin K	Antimicroalgal	*T.* *brevicompactum* A-DL-9-2	Alga *Chondria* *tenuissima*	Dalian, China	[43]
88	Trichodermarin L	Antimicroalgal and antifungal	*T.* *brevicompactum* A-DL-9-2	Alga *Chondria* *tenuissima*	Dalian, China	[43]
89	Trichodermarin M	Antimicroalgal and antifungal	*T.* *brevicompactum* A-DL-9-2	Alga *Chondria* *tenuissima*	Dalian, China	[43]
90	Trichodermarin N		*T.* *brevicompactum* A-DL-9-2	Alga *Chondria* *tenuissima*	Dalian, China	[43]
91	Trichocarotin A		*T.* *virens* Y13-3	Alga *Gracilaria* *vermiculophylla*	Yangma Island, Yantai, China	[44]
92	Trichocarotin B		*T.* *virens* Y13-3	Alga *Gracilaria* *vermiculophylla*	Yangma Island, Yantai, China	[44]
93	Trichocarotin C	Antimicroalgal and zooplankton-toxic	*T.* *virens* Y13-3	Alga *Gracilaria* *vermiculophylla*	Yangma Island, Yantai, China	[44]
94	Trichocarotin D	Antimicroalgal	*T.* *virens* Y13-3	Alga *Gracilaria* *vermiculophylla*	Yangma Island, Yantai, China	[44]
95	Trichocarotin E	Antimicroalgal	*T.* *virens* Y13-3	Alga *Gracilaria* *vermiculophylla*	Yangma Island, Yantai, China	[44]
96	Trichocarotin F		*T.* *virens* Y13-3	Alga *Gracilaria* *vermiculophylla*	Yangma Island, Yantai, China	[44]
97	Trichocarotin G		*T.* *virens* Y13-3	Alga *Gracilaria* *vermiculophylla*	Yangma Island, Yantai, China	[44]
98	Trichocarotin H	Antimicroalgal and zooplankton-toxic	*T.* *virens* Y13-3	Alga *Gracilaria* *vermiculophylla*	Yangma Island, Yantai, China	[44]
99	14-*O*-Methyltrichocarotin G	Antimicroalgal	*T.* *virens* RR-dl-6-8	Alga *Rhodomela* *confervoides*	Dalian, China	[45]
100	14-*O*-Methyl CAF-603	Antimicroalgal	*T.* *virens* RR-dl-6-8	Alga *Rhodomela* *confervoides*	Dalian, China	[45]
101	Trichocadinin A	Zooplankton-toxic	*T.* *virens* Y13-3	Alga *Gracilaria* *vermiculophylla*	Yangma Island, Yantai, China	[44]
102	4-Cadinen-11,12-diol	Antimicroalgal and antibacterial	*T.* *asperellum* A-YMD-9-2	Alga *Gracilaria* *verrucosa*	Yangma Island, Yantai, China	[46]
103	4-Cadinen-11,13-diol	Antimicroalgal and antibacterial	*T.* *asperellum* A-YMD-9-2	Alga *Gracilaria* *verrucosa*	Yangma Island, Yantai, China	[46]
104	Cadin-4-en-11-ol	Antimicroalgal and antibacterial	*T.* *asperelloides* RR-dl-6-11	Alga *Rhodomela* *confervoides*	Dalian, China	[34]
105	Trichocadinin K	Antimicroalgal	*T.* *virens* RR-dl-6-8	alga *Rhodomela* *confervoides*	Dalian, China	[45]
106	Trichocadinin L	Antimicroalgal	*T.* *virens* RR-dl-6-8	Alga *Rhodomela* *confervoides*	Dalian, China	[45]
107	Trichocadinin M	Antimicroalgal	*T.* *virens* RR-dl-6-8	Alga *Rhodomela* *confervoides*	Dalian, China	[45]
108	Trichocadinin N	Antimicroalgal	*T.* *virens* RR-dl-6-8	Alga *Rhodomela* *confervoides*	Dalian, China	[45]
109	Trichodermaloid A	Cytotoxic	*Trichoderma* sp. SM16	Sponge *Dysidea* sp.	Xisha Islands, China	[47]
110	Trichodermaloid B	Cytotoxic	*Trichoderma* sp. SM16	Sponge *Dysidea* sp.	Xisha Islands, China	[47]
111	Trichodermaloid C	Cytotoxic	*Trichoderma* sp. SM16	Sponge *Dysidea* sp.	Xisha Islands, China	[47]
112	Trichocadinin I	Antimicroalgal	*T.* *virens* RR-dl-6-8	Alga *Rhodomela* *confervoides*	Dalian, China	[45]
113	Trichocadinin J	Antimicroalgal	*T.* *virens* RR-dl-6-8	Alga *Rhodomela* *confervoides*	Dalian, China	[45]
114	Trichocadinin H	Antimicroalgal	*T.* *virens* RR-dl-6-8	Alga *Rhodomela* *confervoides*	Dalian, China	[45]
115	Methylhydroheptelidate	Antimicroalgal	*T.* *virens* RR-dl-6-8	Alga *Rhodomela* *confervoides*	Dalian, China	[45]
116	Ethyl hydroheptelidate	Antibacterial and antifungal	*T.* *harzianum* R1	Mangrove *Myoporum* *bontioides*	Leizhou Peninsula, China	[48]
117	8-Acoren-3,11-diol	Antimicroalgal and antibacterial	*T.* *harzianum* X-5	Alga *Laminaria* *japonica*	Chang Islands, China	[30]
118	Trichoacorin A	Antimicroalgal	*T.* *brevicompactum* A-DL-9-2	Alga *Chondria* *tenuissima*	Dalian, China	[37]
119	Trichoacorside A	Antibacterial and antifungal	*T.* *longibrachiatum* EN-586	Alga *Laurencia* *obtusa*	Qingdao, China	[49]
120	Trichocuparin A		*T.* *brevicompactum* A-DL-9-2	Alga *Chondria* *tenuissima*	Dalian, China	[43]
121	Trichocuparin B		*T.* *brevicompactum* A-DL-9-2	Alga *Chondria* *tenuissima*	Dalian, China	[43]
122/123	Trichonerolins A/B	Antimicroalgal	*T.* *brevicompactum* A-DL-9-2	Alga *Chondria* *tenuissima*	Dalian, China	[37]
124	Trichodermoside	Cytotoxic	*Trichoderma* sp. PT2	Alga *Blidingia* *minina*	Wuyu Island, China	[50]
125	Trichodermene A	Antifungal	*T.* *longibrachiatum*	Halophile plant *Suaeda* *glauca*	intertidal zone of Jiaozhou Bay, Qingdao, China	[51]
126	Harzianoic acid A	Antiviral	*T.* *harzianum* LZDX-32-08	Sponge *Xestospongia* *testudinaria*	near Leizhoudao Island, China	[52]
127	Harzianoic acid B	Antiviral	*T.* *harzianum* LZDX-32-08	Sponge *Xestospongia* *testudinaria*	near Leizhoudao Island, China	[52]
128	Trichaspin	Zooplankton-toxic	*T.* *asperellum* cf44-2	Alga *Sargassum* sp.	Zhoushan Islands, China	[26]
129	Harzianone	Antimicroalgal, antibacterial, and zooplankton-toxic	*T.* *longibrachiatum* cf-11	Alga *Codium* *fragile*	coast of Yantai, China	[53, 54]
130	3*S*-Hydroxyharzianone	Antimicroalgal and antibacterial	*T.* *asperellum* A-YMD-9-2	Alga *Gracilaria* *verrucosa*	Yangma Island, Yantai, China	[46]
131	Harziandione A		*T.* *erinaceum* F1-1	Sea star *Acanthaster* *planci*	Hainan Sanya National Coral Reef Reserve, China	[32]
132	Harzianol K	Anti-inflammatory	*Trichoderma* sp. SCSIOW21	Sediment	South China Sea (-2134 m), China	[55]
133	Harzianol L	Anti-inflammatory	*Trichoderma* sp. SCSIOW21	Sediment	South China Sea (-2134 m), China	[55]
134	Harzianol M		*Trichoderma* sp. SCSIOW21	Sediment	South China Sea (-2134 m), China	[55]
135	Harzianol N		*Trichoderma* sp. SCSIOW21	Sediment	South China Sea (-2134 m), China	[55]
136	Harzianol O		*Trichoderma* sp. SCSIOW21	Sediment	South China Sea (-2134 m), China	[55]
137	Harzianone E	Antibacterial	*T.* *harzianum* XS-20090075	Unidentified soft coral	Xisha Islands, China	[56]
138	Harzianone A	Phytotoxic	*T.* *harzianum* XS-20090075	Unidentified soft coral	Xisha Islands, China	[57]
139	Harzianone B	Phytotoxic	*T.* *harzianum* XS-20090075	Unidentified soft coral	Xisha Islands, China	[57]
140	Harzianone C	Phytotoxic	*T.* *harzianum* XS-20090075	Unidentified soft coral	Xisha Islands, China	[57]
141	Harzianone D		*T.* *harzianum* XS-20090075	Unidentified soft coral	Xisha Islands, China	[57]
142	Harziane	Phytotoxic	*T.* *harzianum* XS-20090075	Unidentified soft coral	Xisha Islands, China	[57]
143	11-Hydroxy-9-harzien-3-one	Antibacterial and zooplankton-toxic	*T.* *asperellum* cf44-2	Alga *Sargassum* sp.	Zhoushan Islands, China	[26]
144	(9*R*,10*R*)-Dihydro-harzianone	Cytotoxic	*Trichoderma* sp. Xy24	Mangrove *Xylocarpus* *granatum*	Sanya district, Hainan province, China	[58]
145	3*R*-Hydroxy-9*R*,10*R*-dihydroharzianone	Antimicroalgal and antibacterial	*T.* *harzianum* X-5	Alga *Laminaria* *japonica*	Chang Islands, China	[30]
146	3*S*-Hydroxy-9*R*,10*R*-dihydroharzianone	Antimicroalgal	*T.* *asperelloides* RR-dl-6-11	Alga *Rhodomela* *confervoides*	Dalian, China	[59]
147	Harzianelactone		*Trichoderma* sp. Xy24	Mangrove *Xylocarpus* *granatum*	Sanya district, Hainan province, China	[58]
148	Harzianelactone A	Phytotoxic	*T.* *harzianum* XS-20090075	Unidentified soft coral	Xisha Islands, China	[57]
149	Deoxytrichodermaerin	Antimicroalgal and zooplankton-toxic	*T.* *longibrachiatum* A-WH-20-2	Alga *Laurencia* *okamurai*	Weihai, China	[54]
150	Trichodermaerin		*T.* *erinaceum* 2011F1-1 (or F1-1)	Sea star *Acanthaster* *planci*	Hainan Sanya National Coral Reef Reserve, China	[60]
151	Harzianelactone B	Phytotoxic	*T.* *harzianum* XS-20090075	Unidentified soft coral	Xisha Islands, China	[57]
152	3*S*-Hydroxytrichodermaerin	Antimicroalgal	*T.* *asperelloides* RR-dl-6-11	Alga *Rhodomela* *confervoides*	Dalian, China	[59]
153	Methyl 3*S*-hydroxy-10,11-*seco*-harzianate	Antimicroalgal	*T.* *asperelloides* RR-dl-6-11	Alga *Rhodomela* *confervoides*	Dalian, China	[59]
154	11*R*-Methoxy-5,9,13-proharzitrien-3-ol	Antimicroalgal and antibacterial	*T.* *harzianum* X-5	Alga *Laminaria* *japonica*	Chang Islands, China	[30]
155	11*R*-Methoxy-5,9,13-proharzitrien-19-ol	Antimicroalgal	*T.* *asperelloides* RR-dl-6-11	Alga *Rhodomela* *confervoides*	Dalian, China	[59]
156	Trichodermanin C	Cytotoxic	*T.* *harzianum* OUPS-111D-4	Sponge *Halichondria* *okadai*	Osaka bay, Japan	[61]
157	Trichodermanin D	Cytotoxic	*T.* *harzianum* OUPS-111D-4	Sponge *Halichondria* *okadai*	Osaka bay, Japan	[61]
158	Trichodermanin E	Cytotoxic	*T.* *harzianum* OUPS-111D-4	Sponge *Halichondria* *okadai*	Osaka bay, Japan	[61, 62]
159	Trichodermanin F	Cytotoxic	*T.* *harzianum* OUPS-111D-4	Sponge *Halichondria* *okadai*	Osaka bay, Japan	[62]
160	Trichodermanin G		*T.* *harzianum* OUPS-111D-4	Sponge *Halichondria* *okadai*	Osaka bay, Japan	[62]
161	Trichodermanin H	Cytotoxic	*T.* *harzianum* OUPS-111D-4	Sponge *Halichondria* *okadai*	Osaka bay, Japan	[62]
162	Citrinovirin	Antibacterial, antimicroalgal, and zooplankton-toxic	*T.* *citrinoviride* cf-27	Alga *Dictyopteris* *prolifera*	Zhoushan Islands, China	[63]
163	Trichocitrin	Antimicroalgal and antibacterial	*T.* *citrinoviride* cf-27	Alga *Dictyopteris* *prolifera*	Zhoushan Islands, China	[64]
164	Harzianolic acid A		*T.* *harzianum* XS-20090075	Unidentified soft coral	Xisha Islands, China	[56]
165	Trichosordarin A	Antimicroalgal and zooplankton-toxic	*T.* *harzianum* R5	Sediment	Bohai Sea, China	[65]
166	Isoergokonin B	Antimicroalgal and antibacterial	*T.* *brevicompactum* A-DL-9-2	Alga *Chondria* *tenuissima*	Dalian, China	[37]
167	Tricholumin A	Antimicroalgal, antibacterial, and antifungal	*T.* *asperellum* cf44-2	Alga *Sargassum* sp.	Zhoushan Islands, China	[66]
168	4-(*p*-Hydroxyphenethoxy)demethylincisterol A_3_	Antimicroalgal and antibacterial	*T.* *atroviride* RR-dl-3-9	Alga *Rhodomela* *confervoides*	Dalian, China	[39]

**Table 2:** Polyketides (**169**–**295**) from the marine-derived *Trichoderma*

No.	Name	Bioactivity	Producer	Source	Locality	References
169	Harzianumol A		*T.* *harzianum* HMS-15–3 (or HNS-15–3)	Sponge *Petrospongia* *nigra*	South China Sea, China	[67]
170	Harzianumol B		*T.* *harzianum* HMS-15–3 (or HNS-15–3)	Sponge *Petrospongia* *nigra*	South China Sea, China	[67]
171	Harzianumol C		*T.* *harzianum* HMS-15–3 (or HNS-15–3)	Sponge *Petrospongia* *nigra*	South China Sea, China	[67]
172	Harzianumol D		*T.* *harzianum* HMS-15–3 (or HNS-15–3)	Sponge *Petrospongia* *nigra*	South China Sea, China	[67]
173	Harzianumol E		*T.* *harzianum* HMS-15–3 (or HNS-15–3)	Sponge *Petrospongia* *nigra*	South China Sea, China	[67]
174	Harzianumol F		*T.* *harzianum* HMS-15–3 (or HNS-15–3)	Sponge *Petrospongia* *nigra*	South China Sea, China	[67]
175	Harzianumol G		*T.* *harzianum* HMS-15–3 (or HNS-15–3)	Sponge *Petrospongia* *nigra*	South China Sea, China	[67]
176	Harzianumol H		*T.* *harzianum* HMS-15–3 (or HNS-15–3)	Sponge *Petrospongia* *nigra*	South China Sea, China	[67]
177	Methyl 3,5-dihydroxydodecanoate	Antimicroalgal and antibacterial	*T.* *atroviride* RR-dl-3–9	Alga *Rhodomela* *confervoides*	Dalian, China	[39]
178	(2S*,3S*,5R*,8*E*)-2-Methyl-8-decene-1,3,5-triol	Antimicroalgal and antibacterial	*T.* *citrinoviride* A-WH-20–3	Alga *Laurencia* *okamurai*	Weihai, China	[68]
179	Citrinoviric acid	Cytotoxic	*T.* *citrinoviride*	Sediment	Langqi Island, Fujian, China	[69]
180	Nafuredin C	Antifungal	*T.* *harzianum* D13	Mangrove *Excoecaria* *agallocha*	Hainan province, China	[70]
181	Harzialactone B	Cytotoxic	*T.* *harzianum* OUPS-N 115	Sponge *Halichondria* *okadai*	Tanabe Bay, Japan	[71]
182	Trichodenone A	Cytotoxic	*T.* *harzianum* OUPS-N 115	Sponge *Halichondria* *okadai*	Tanabe Bay, Japan	[71, 72]
183	Trichodenone B	Cytotoxic	*T.* *harzianum* OUPS-N 115	Sponge *Halichondria* *okadai*	Tanabe Bay, Japan	[71, 72]
184	Trichodenone C	Cytotoxic	*T.* *harzianum* OUPS-N 115	Sponge *Halichondria* *okadai*	Tanabe Bay, Japan	[71, 72]
185	Trichoderone	Cytotoxic	*Trichoderma* sp. GIBH-Mf082	Deep sea sediment	South China Sea, China	[73]
186	Dechlorotrichodenone C	Antimicroalgal and antibacterial	*T.* *asperellum* cf44-2	Alga *Sargassum* sp.	Zhoushan Islands, China	[25]
187	3-Hydroxytrichodenone C	Antimicroalgal and antibacterial	*T.* *asperellum* cf44-2	Alga *Sargassum* sp.	Zhoushan Islands, China	[25]
188	Hypocnone A		*H.* *koningii* (*T.* *koningii*)	Sponge *Haliclona* sp.	Sanya, Hainan Island, China	[74]
189	Methyl 3-(3-oxocyclopent-1-enyl)propionate		*T.* *atroviride* G20-12	Sediment on the root of mangrove *Ceriops* *tagal*	South China Sea, China	[75]
190	Trichodermacid A		*T.* *atroviride* H548	Sediment	Fujian province, China	[76]
191	Trichodermester A	Antifungal	*T.* *atroviride* H548	Sediment	Fujian province, China	[76]
192	Trichodermester B		*T.* *atroviride* H548	Sediment	Fujian province, China	[76]
193	Hypocrenone A		*H.* *koningii* (*T.* *koningii*) PF04	Sponge *Phakellia* *fusca*	Yongxing Island in the South China Sea, China	[77]
194	Hypocrenone B		*H.* *koningii* (*T.* *koningii*) PF04	Sponge *Phakellia* *fusca*	Yongxing Island in the South China Sea, China	[77]
195	Hypocrenone C		*H.* *koningii* (*T.* *koningii*) PF04	Sponge *Phakellia* *fusca*	Yongxing Island in the South China Sea, China	[77]
196	5-Hydroxycyclopeni cillone	Antioxidant, anti-Aβ fibrillization and neuroprotective	*Trichoderma* sp. HPQJ-34	Sponge *Hymeniacidon* *perleve*	Dongji Island, Zhejiang province, China	[78]
197	6-Demethyl-sorbicillin	Cytotoxic	*Trichoderma* sp. f-13	Sediment	Fujian province,China	[79]
198	(4′*Z*)-Sorbicillin		*Trichoderma* sp. AF007	Sea star *Acanthaster* *planci*	Hainan Sanya National Coral Reef Reserve, China	[80]
199	(2*S*)-2,3-Dihydro-7-hydroxy-6-methyl-2-[(*E*)-prop-1-enyl]-chroman-4-one	Cytotoxic	*Trichoderma* sp. AF007	Sea star *Acanthaster* *planci*	Hainan Sanya National Coral Reef Reserve, China	[80]
200	Trichosorbicillin B	Anti-inflammatory	*T.* *reesei* 4670	Sponge	Shantou, Guangdong Province, China	[81]
201	Trichosorbicillin C	Anti-inflammatory	*T.* *reesei* 4670	Sponge	Shantou, Guangdong Province, China	[81]
202	Trichosorbicillin D		*T.* *reesei* 4670	Sponge	Shantou, Guangdong Province, China	[81]
203	12-Hydroxysorbicillin	Anti-inflammatory	*T.* *reesei* 4670	Sponge	Shantou, Guangdong Province, China	[81]
204	8,9-Dihydro-12-hydroxysorbicillin	Anti-inflammatory	*T.* *reesei* 4670	Sponge	Shantou, Guangdong Province, China	[81]
205	Trichosorbicillin E	Anti-inflammatory	*T.* *reesei* 4670	Sponge	Shantou, Guangdong Province, China	[81]
206	Trichosorbicillin F	Anti-inflammatory	*T.* *reesei* 4670	Sponge	Shantou, Guangdong Province, China	[81]
207	Trichosorbicillin G		*T.* *reesei* 4670	Sponge	Shantou, Guangdong Province, China	[81]
208	Isotrichosorbicillin E	Anti-inflammatory	*T.* *reesei* 4670	Sponge	Shantou, Guangdong Province, China	[81]
209	Trichosorbicillin H		*T.* *reesei* 4670	Sponge	Shantou, Guangdong Province, China	[81]
210	3-Methyltrichosorbicillin H		*T.* *reesei* 4670	Sponge	Shantou, Guangdong Province, China	[81]
211	Trichosorbicillin I	Anti-inflammatory	*T.* *reesei* 4670	Sponge	Shantou, Guangdong Province, China	[81]
212	1-(2,4-Dihydroxy-3,5-dimethylphenyl)-3,4,5-trihydroxyhexan-1-one		*H.* *jecorina* (*T.* *reesei*) H8	Mangrove sediment	Fujian province, China	[82]
213	Sorbicilliside B	Antibacterial and antifungal	*T.* *longibrachiatum* EN-586	Alga *Laurencia* *obtusa*	Qingdao, China	[49]
214	Sorbicilliside A	Antibacterial and antifungal	*T.* *longibrachiatum* EN-586	Alga *Laurencia* *obtusa*	Qingdao, China	[49]
215	(−)-Trichodermatone	Cytotoxic	*Trichoderma* sp. FM652	Sediment	Hawaii, USA	[83]
216	Saturnispol E		*T.* *saturnisporum* DI-IA	Sponge *Dictyonella* *incisa*	Seferihisar bay, Turkey	[84]
217	Epoxysorbicillinol		*T.* *longibrachiatum*	Sponge *Haliclona* sp.		[85]
218	24-Hydroxy-trichodimerol	Cytotoxic	*T.* *reesei* HN-2016–018	Unidentified sponge	South China Sea, China	[86]
219	7,7′,9′-Hydroxy-trichodimerol		*H.* *jecorina* (*T.* *reesei*) H8	Mangrove sediment	Fujian province, China	[82]
220	Isobisvertinol A	Antifungal and zebrafish-toxic	*H.* *jecorina* (*T.* *reesei*) H8	Mangrove sediment	Fujian province, China	[82]
221	15-Hydroxy-bisvertinol		*T.* *reesei* HN-2016–018	Unidentified sponge	South China Sea, China	[86]
222	Saturnispol A		*T.* *saturnisporum* DI-IA	Sponge *Dictyonella* *incisa*	Seferihisar bay, Turkey	[84]
223	Saturnispol B	Anti-inflammatory	*T.* *saturnisporum* DI-IA	Sponge *Dictyonella* *incisa*	Seferihisar bay, Turkey	[81, 84]
224	Trichobisvertinol A	Anti-inflammatory	*T.* *reesei* 4670	Sponge	Shantou, Guangdong Province, China	[81]
225	Trichobisvertinol B	Anti-inflammatory	*T.* *reesei* 4670	Sponge	Shantou, Guangdong Province, China	[81]
226	Trichobisvertinol C	Anti-inflammatory	*T.* *reesei* 4670	Sponge	Shantou, Guangdong Province, China	[81]
227	Trichobisvertinol D	Anti-inflammatory	*T.* *reesei* 4670	Sponge	Shantou, Guangdong Province, China	[81]
228	12-*Epi*-trichobisvertinol D	Anti-inflammatory	*T.* *reesei* 4670	Sponge	Shantou, Guangdong Province, China	[81]
229	10,11-Dihydrobisvertinolone	Cytotoxic	*Trichoderma* sp. f-13	Sediment	Fujian province, China	[79]
230	2,3-Dihydro 2-hydroxy vertinolide	Cytotoxic and NF-κB-inhibitory	*Trichoderma* sp. FM652	Sediment	Hawaii, USA	[83]
231	Saturnispol F	Antibacterial	*T.* *saturnisporum* DI-IA	Sponge *Dictyonella* *incisa*	Seferihisar bay, Turkey	[84]
232	Saturnispol C		*T.* *saturnisporum* DI-IA	Sponge *Dictyonella* *incisa*	Seferihisar bay, Turkey	[84]
233	Saturnispol D		*T.* *saturnisporum* DI-IA	Sponge *Dictyonella* *incisa*	Seferihisar bay, Turkey	[84]
234	Trichodermanone A	Antioxidant	*Trichoderma* sp.	Sponge *Agelas* *dispar*	Island of Dominica	[87]
235	Trichodermanone B	Antioxidant	*Trichoderma* sp.	Sponge *Agelas* *dispar*	Island of Dominica	[87]
236	Trichodermanone C	Antioxidant, anti-inflammatory	*Trichoderma* sp.	Sponge *Agelas* *dispar*	Island of Dominica	[87, 88]
237	Trichodermanone D		*Trichoderma* sp.	Sponge *Agelas* *dispar*	Island of Dominica	[87]
238	Trichoreeseione A		*T.* *reesei* HN-2016-018	Unidentified sponge	South China Sea, China	[86]
239	Trichoreeseione B		*T.* *reesei* HN-2016-018	Unidentified sponge	South China Sea, China	[86]
240	Trichodermolide B		*T.* *reesei* HN-2016-018	Unidentified sponge	South China Sea, China	[86]
241	13-Hydroxy-trichodermolide		*T.* *reesei* HN-2016-018	Unidentified sponge	South China Sea, China	[86]
242	Trichodermolide C		*H.* *jecorina* (*T.* *reesei*) H8	Mangrove sediment	Fujian province, China	[82]
243	Trichodermolide D		*H.* *jecorina* (*T.* *reesei*) H8	Mangrove sediment	Fujian province, China	[82]
244	Trichodermatide A	Cytotoxic	*T.* *reesei* YZ48-08	Mud in the tideland	Lianyungang, China	[89]
245	Trichodermatide B	Cytotoxic	*T.* *reesei* YZ48-08	Mud in the tideland	Lianyungang, China	[89]
246	Trichodermatide C	Cytotoxic	*T.* *reesei* YZ48-08	Mud in the tideland	Lianyungang, China	[89]
247	Trichodermatide D	Cytotoxic	*T.* *reesei* YZ48-08	Mud in the tideland	Lianyungang, China	[89]
248	4-(5,7-Dimethoxy-4-oxo-4*H*-chromen-2-yl)heptanoic acid methyl ester		*H.* *lixii* SCSIO 41520	Unidentified soft coral	Daya Bay, Shenzhen, China	[90]
249	7-*O*-Methylkoninginin D		*T.* *koningii* MF349	Mud	South China Sea, China	[91]
250	Trichodermaketone A	Synergistic antifungal	*T.* *koningii* MF349	Mud	South China Sea, China	[91]
251	Trichodermaketone B		*T.* *koningii* MF349	Mud	South China Sea, China	[91]
252	Trichodermaketone C		*T.* *koningii* MF349	Mud	South China Sea, China	[91]
253	Trichodermaketone D		*T.* *koningii* MF349	Mud	South China Sea, China	[91]
254	Trichoketide A	Enzyme-inhibitory	*Trichoderma* sp. TPU1237	Seawater	Ashizaki-Bay in Mutsu city, Japan	[92]
255	Trichoketide B	Enzyme-inhibitory	*Trichoderma* sp. TPU1237	Seawater	Ashizaki-Bay in Mutsu city, Japan	[92]
256	Trichoharzianin		*T.* *harzianum* PSU-MF79	Unidentified tunicate	Phuket Province, Thailand	[93]
257	Tandyukisin G		*Trichoderma* sp. JWM29-10–1	Hydrothermal vent sediment	Kueishantao, China	[94]
258	Tandyukisin H		*Trichoderma* sp. JWM29-10–1	Hydrothermal vent sediment	Kueishantao, China	[94]
259	Tandyukisin I		*Trichoderma* sp. JWM29-10–1	Hydrothermal vent sediment	Kueishantao, China	[94]
260	Trichoharzin		*T.* *harzianum*	Sponge *Micale* *cecilia*	Amami Island, Japan	[24]
261	Tandyukisin (or tandyukisin A)	Cytotoxic	*T.* *harzianum* OUPS-111D-4	Sponge *Halichondria* *okadai*	Osaka bay, Japan	[95]
262	Tandyukisin B	Cytotoxic	*T.* *harzianum* OUPS-111D-4	Sponge *Halichondria* *okadai*	Osaka bay, Japan	[96]
263	Tandyukisin C	Cytotoxic	*T.* *harzianum* OUPS-111D-4	Sponge *Halichondria* *okadai*	Osaka bay, Japan	[96]
264	Tandyukisin D	Cytotoxic	*T.* *harzianum* OUPS-111D-4	Sponge *Halichondria* *okadai*	Osaka bay, Japan	[96]
265	Tandyukisin E	Cytotoxic	*T.* *harzianum* OUPS-111D-4	Sponge *Halichondria* *okadai*	Osaka bay, Japan	[97]
266	Tandyukisin F	Cytotoxic	*T.* *harzianum* OUPS-111D-4	Sponge *Halichondria* *okadai*	Osaka bay, Japan	[97]
267	Trichoharzin B		*T.* *harzianum* XS-20090075	Unidentified soft coral	Xisha Islands, China	[98]
268	Methyl-trichoharzin	Antifouling	*T.* *harzianum* XS-20090075	Unidentified soft coral	Xisha Islands, China	[98]
269	Trichodermaxanthone		*T.* *aureoviride* PSU-F95	Sea fan *Annella* sp.	Similan Islands, Thailand	[99]
270	Trichodermaquinone		*T.* *aureoviride* PSU-F95	Sea fan *Annella* sp.	Similan Islands, Thailand	[99]
271	Harzianumnone A		*T.* *harzianum* XS-20090075	Soft coral	Xisha Islands coral reef in the South China Sea, China	[100]
272	Harzianumnone B		*T.* *harzianum* XS-20090075	Soft coral	Xisha Islands coral reef in the South China Sea, China	[100]
273	7-Acetyl-1,3,6-trihydroxyanthracene-9,10- dione		*Trichoderma* sp. SCSIO41004	Sponge *Callyspongia* sp.	near Xuwen County, Guangdong Province, China	[101]
274	5,7-Dihydroxy-3-methyl-2-(2-oxopropyl)naphthalene-1,4-dione		*Trichoderma* sp. SCSIO41004	Sponge *Callyspongia* sp.	near Xuwen County, Guangdong Province, China	[101]
275	Trichbenzoisochromen A		*Trichoderma* sp. SCSIO41004	Sponge *Callyspongia* sp.	near Xuwen County, Guangdong Province, China	[101]
276	Hypochromin A	Enzyme-inhibitory and cytotoxic	*H.* *vinosa*	Sediment (beach sand)	Okinawa Prefecture, Japan	[102]
277	Hypochromin B	Enzyme-inhibitory and cytotoxic	*H.* *vinosa*	Sediment (beach sand)	Okinawa Prefecture, Japan	[102]
278	Trichorenin A	Antimicroalgal	*T.* *virens* Y13-3	Alga *Gracilaria* *vermiculophylla*	Yangma Island, Yantai, China	[103]
279	Trichorenin B	Antimicroalgal	*T.* *virens* Y13-3	Alga *Gracilaria* *vermiculophylla*	Yangma Island, Yantai, China	[103]
280	Trichorenin C	Antimicroalgal	*T.* *virens* Y13-3	Alga *Gracilaria* *vermiculophylla*	Yangma Island, Yantai, China	[103]
281	Harzialactone A	Cytotoxic	*T.* *harzianum* OUPS-N 115	Sponge *Halichondria* *okadai*	Tanabe Bay, Japan	[71, 104]
282	3-Hydroxy-5-(4-hydroxybenzyl)dihydrofuran-2(3*H*)-one		*T.* *atroviride* G20-12	Sediment on the root of mangrove *Ceriops* *tagal*	South China Sea, China	[105]
283	Trichoderolide C		*T.* *erinaceum* F1-1	Sea star *Acanthaster* *planci*	Hainan Sanya National Coral Reef Reserve, China	[32]
284	Trichoderolide D		*T.* *erinaceum* F1-1	Sea star *Acanthaster* *planci*	Hainan Sanya National Coral Reef Reserve, China	[32]
285	Trichoderolide E		*T.* *erinaceum* F1-1	Sea star *Acanthaster* *planci*	Hainan Sanya National Coral Reef Reserve, China	[32]
286	Trichoderolide F		*T.* *erinaceum* F1-1	Sea star *Acanthaster* *planci*	Hainan Sanya National Coral Reef Reserve, China	[32]
287	Trichoderolide A		*T.* *erinaceum* F1-1	Sea star *Acanthaster* *planci*	Hainan Sanya National Coral Reef Reserve, China	[32]
288	Trichoderolide B	Cytotoxic	*T.* *erinaceum* F1-1	Sea star *Acanthaster* *planci*	Hainan Sanya National Coral Reef Reserve, China	[32]
289	Trichophenol A	Antimicroalgal and antibacterial	*T.* *citrinoviride* A-WH-20–3	Alga *Laurencia* *okamurai*	Weihai, China	[33]
290	(2*E*)-1-[(5-Hydroxy-7-methoxy-2-methyl-4-oxo-4*H*-1-benzopyran-3-yl)methyl]3-methyl-2-pentenedioate	Antibacterial and antifungal	*Trichoderma* sp. JWM29-10–1	Hydrothermal vent sediment	Kueishantao, China	[94]
291	(2*S*,3*S*)-5-Hydroxy-3-hydroxymethyl-7-methoxy-2-methyl-4-chromanone	Antibacterial	*Trichoderma* sp. JWM29-10–1	Hydrothermal vent sediment	Kueishantao, China	[94]
292	Trichoharzianone		*T.* *harzianum* PSU-MF79	Unidentified tunicate	Phuket Province, Thailand	[93]
293	Trichopyrone		*T.* *viride*	Sponge *Agelas* *dispar*	Island of Dominica	[106]
294	Saturnispol G		*T.* *saturnisporum* DI-IA	Sponge *Dictyonella* *incisa*	Seferihisar bay, Turkey	[84]
295	Saturnispol H	Antibacterial	*T.* *saturnisporum* DI-IA	Sponge *Dictyonella* *incisa*	Seferihisar bay, Turkey	[84]

**Table 3:** Peptides (**296**–**426**), alkaloids (**427**–**440**), and others (**441**–**445**) from the marine-derived *Trichoderma*

No.	Name	Bioactivity	Producer	Source	Locality	Reference
296	DC1149R		*Trichoderma* sp. TPU199 (cf. *T.* *brevicompactum*)	Unidentified alga	Palau	[107]
297	Iododithiobrevamide		*Trichoderma* sp. TPU199 (cf. *T.* *brevicompactum*)	Unidentified alga	Palau	[107]
298	5-*epi*-Pretrichodermamide A		*Trichoderma* sp. TPU199 (cf. *T.* *brevicompactum*)	Unidentified alga	Palau	[41]
299	5-*epi*-Trithiopretrichodermamide A		*Trichoderma* sp. TPU199 (cf. *T.* *brevicompactum*)	Unidentified alga	Palau	[41]
300	Chlorotrithiobrevamide		*Trichoderma* sp. TPU199 (cf. *T.* *brevicompactum*)	Unidentified alga	Palau	[108]
301	Dehydroxymethylbis(dethio)bis(methylthio)gliotoxin		*T.* *virens* Y13-3	Alga *Gracilaria* *vermiculophylla*	Yangma Island, Yantai, China	[109]
302	(3*S*,6*R*)-6-(Para-hydroxybenzyl)-1,4-dimethyl-3,6-bis(methylthio)piperazine-2,5-dione		*T.* *virens* Y13-3	Alga *Gracilaria* *vermiculophylla*	Yangma Island, Yantai, China	[109]
303	Methylcordysinin A		*T.* *asperellum* cf44-2	Alga *Sargassum* sp.	Zhoushan Islands, China	[25]
304	Cyclo(L-5-MeO-Pro-L-5-MeO-Pro)	Antimicroalgal	*T.* *asperellum* A-YMD-9–2	Alga *Gracilaria* *verrucosa*	Yangma Island, Yantai, China	[35]
305	Atroviridetide		*T.* *atroviride* G20-12	Sediment on the root of mangrove *Ceriops* *tagal*	South China Sea, China	[110]
306	Trichoderide A	Cytotoxic	*T.* *reesei* YZ48-08	Mud in the tideland	Lianyungang, China	[111]
307	Trichodermamide A		*T.* *virens* CNL910	Ascidian *Didemnum* *molle*	near Madang, Papua New Guinea	[112]
308	Trichodermamide B	Antibacterial, antifungal, cytotoxic	*T.* *virens* CNL910 & CNK266	Ascidian *Didemnum* *molle* & alga *Halimeda* sp.	near Madang, Papua New Guinea	[112]
309	Trichodermamide G		*T.* *harzianum* D13	Mangrove *Excoecaria* *agallocha*	Hainan province, China	[70]
310	Dithioaspergillazine A	Cytotoxic	*Trichoderma* sp. TPU199 (cf. *T.* *brevicompactum*)	Unidentified alga	Palau	[113]
311	Trichoderin A	Antibacterial	*Trichoderma* sp. 05FI48	Unidentified sponge		[114, 115]
312	Trichoderin A1	Antibacterial	*Trichoderma* sp. 05FI48	Unidentified sponge		[114]
313	Trichoderin B	Antibacterial	*Trichoderma* sp. 05FI48	Unidentified sponge		[114]
314	Aspereline A		*T.* *asperellum* Y19-07 (or Y19-17)	Sediment	Antarctic Penguin Island	[116]
315	Aspereline B		*T.* *asperellum* Y19-07 (or Y19-17)	Sediment	Antarctic Penguin Island	[116]
316	Aspereline C		*T.* *asperellum* Y19-07 (or Y19-17)	Sediment	Antarctic Penguin Island	[116]
317	Aspereline D		*T.* *asperellum* Y19-07 (or Y19-17)	Sediment	Antarctic Penguin Island	[116]
318	Aspereline E		*T.* *asperellum* Y19-07 (or Y19-17)	Sediment	Antarctic Penguin Island	[116]
319	Aspereline F		*T.* *asperellum* Y19-07 (or Y19-17)	Sediment	Antarctic Penguin Island	[116]
320	Aspereline G		*T.* *asperellum*	Sediment	Langqi Island, Fujian, China	[117]
321	Aspereline H		*T.* *asperellum*	Sediment	Langqi Island, Fujian, China	[117]
322–353	Asperelines G-U, W-Z, and Z_1_-Z_13_		*T.* *asperellum* Y19-17 (or Y19-07)	Sediment	Antarctic Penguin Island	[118]
354	Trichobrachin A I		*T.* *longibrachiatum* MMS 151	Mussel *Mytilus* *edulis*	Tharon, France	[119]
355	Trichobrachin A II		*T.* *longibrachiatum* MMS 151	Mussel *Mytilus* *edulis*	Tharon, France	[119]
356	Trichobrachin A III		*T.* *longibrachiatum* MMS 151	Mussel *Mytilus* *edulis*	Tharon, France	[119]
357	Trichobrachin A IV		*T.* *longibrachiatum* MMS 151	Mussel *Mytilus* *edulis*	Tharon, France	[119]
358	Trichobrachin B I		*T.* *longibrachiatum* MMS 151	Mussel *Mytilus* *edulis*	Tharon, France	[119]
359	Trichobrachin B II		*T.* *longibrachiatum* MMS 151	Mussel *Mytilus* *edulis*	Tharon, France	[119]
360	Trichobrachin B III		*T.* *longibrachiatum* MMS 151	Mussel *Mytilus* *edulis*	Tharon, France	[119]
361	Trichobrachin B IV		*T.* *longibrachiatum* MMS 151	Mussel *Mytilus* *edulis*	Tharon, France	[119]
362–382	Trichobrachin A-IX		*T.* *longibrachiatum* DAOM 234100	Mussel *Mytilus* *edulis*	estuary of the Loire River (Tharon, France)	[120]
383	TA-19A-Ia		*T.* *atroviride* MMS927, MMS639, MMS925, MMS1295, MMS1513	Seawater, sediments, and blue mussels	La Couplasse, Le Croisic, and Bonne Anse	[121]
384	TA-19A-IIa		*T.* *atroviride* MMS927, MMS639, MMS925, MMS1295, MMS1513	Seawater, sediments, and blue mussels	La Couplasse, Le Croisic, and Bonne Anse	[121]
385	TA-19A-IIIa		*T.* *atroviride* MMS927, MMS639, MMS925, MMS1295, MMS1513	Seawater, sediments, and blue mussels	La Couplasse, Le Croisic, and Bonne Anse	[121]
386	Trichorzianine 1938	Antibacterial	*T.* *atroviride* NF16	Sponge *Axinella* sp.	Mediterranean sea, Akhziv, Israel	[122]
387	Trichorzianine 1909	Antibacterial	*T.* *atroviride* NF16	Sponge *Axinella* sp.	Mediterranean sea, Akhziv, Israel	[122]
388	Trichorzianine 1895	Antibacterial	*T.* *atroviride* NF16	Sponge *Axinella* sp.	Mediterranean sea, Akhziv, Israel	[122]
389	Trichorzianine 1896	Antibacterial	*T.* *atroviride* NF16	Sponge *Axinella* sp.	Mediterranean sea, Akhziv, Israel	[122]
390	Trichorzianine 1924	Antibacterial	*T.* *atroviride* NF16	Sponge *Axinella* sp.	Mediterranean sea, Akhziv, Israel	[122]
391	Trichorzianine 1910	Antibacterial	*T.* *atroviride* NF16	Sponge *Axinella* sp.	Mediterranean sea, Akhziv, Israel	[122]
392	Trichorzianine 1924a	Antibacterial	*T.* *atroviride* NF16	sponge *Axinella* sp.	Mediterranean sea, Akhziv, Israel	[122]
393	Trichorzianine 1909a	Antibacterial	*T.* *atroviride* NF16	Sponge *Axinella* sp.	Mediterranean sea, Akhziv, Israel	[122]
394	Longibrachin A-0		*T.* *longibrachiatum* MMS151	Mussel *Mytilus* *edulis*	Estuary of the river Loire, Tharon, France	[123]
395	Longibrachin A-II-a	Cytotoxic, antibacterial, antifungal, and insect-toxic	*T.* *longibrachiatum* MMS151	Mussel *Mytilus* *edulis*	Estuary of the river Loire, Tharon, France	[123]
396	Longibrachin A-IV-b		*T.* *longibrachiatum* MMS151	Mussel *Mytilus* *edulis*	Estuary of the river Loire, Tharon, France	[123]
397	Hyporientalin A	Antibacterial and antifungal	*T.* *orientale* LSBA1	Sponge *Cymbaxinella* *damicornis*	Mediterranean Sea, Mahdia, Tunisia	[124]
398–426	TA-17A-Ix to -IVx and TA-17S-Ix to -IIIx		*T.* *atroviride* MMS927, MMS639, MMS1295	Seawater, sediments, and blue mussels	La Couplasse, Le Croisic, and Bonne Anse	[121]
427	Trichocarboline A	Anti-pulmonary fibrosis	*Trichoderma* sp. MCCC 3A01244	Seawater (deep sea-3300 m)	South China Sea	[125]
428	Trichocarboline C		*Trichoderma* sp. MCCC 3A01244	Seawater (deep sea-3300 m)	South China Sea	[125]
429	(–)-Trichocarboline B	Anti-pulmonary fibrosis	*Trichoderma* sp. MCCC 3A01244	Seawater (deep sea-3300 m)	South China Sea	[125]
430	(+)-Trichocarboline B	Anti-pulmonary fibrosis	*Trichoderma* sp. MCCC 3A01244	Seawater (deep sea-3300 m)	South China Sea	[125]
431	Trichodin A	Antibacterial and antifungal	*Trichoderma* sp. MF106		Greenland Sea (Fram Strait)	[126]
432	Trichodin B		*Trichoderma* sp. MF106		Greenland Sea (Fram Strait)	[126]
433	Ethyl 2-bromo-4-chloroquinoline-3-carboxylate		*T.* *harzianum* XS-20090075	Unidentified soft coral	Xisha Islands, China	[98]
434	2-Methylimidazo[1,5-*b*]isoquinoline-1,3,5(2*H*)-trione		*H.* *virens*	Mangrove *Rhizophora* *apiculata*	Guangxi Province, China	[127]
435	4-Oxazolepropanoic acid		*T.* *asperellum* cf44-2	Alga *Sargassum* sp.	Zhoushan Islands, China	[25]
436	(3′-Hydroxybutan-2′-yl)5-oxopyrrolidine-2-carboxylate		*T.* *atroviride* G20-12	Sediment on the root of mangrove *Ceriops* *tagal*	South China Sea, China	[110]
437	Trichosorbicillin A		*T.* *reesei* 4670	Sponge	Shantou, Guangdong Province, China	[81]
438	Penicillenol F	Cytotoxic	*T.* *citrinoviride*	Sediment	Fuzhou, China	[128]
439	Penicillenol D	Cytotoxic	*T.* *citrinoviride*	Sediment	Langqi Island, Fujian, China	[69]
440	2-Hydroxybutan-3-yl 5′-(2′′-hydroxy-*N*-(2′′′-oxobutan-3′′′-yl)propanamido)butanoate		*T.* *atroviride* G20-12	Sediment on the root of mangrove *Ceriops* *tagal*	South China Sea, China	[105]
441	4′-(4,5-Dimethyl-1,3-dioxolan-2-yl)methyl-phenol		*T.* *atroviride* G20-12	Sediment on the root of mangrove *Ceriops* *tagal*	South China Sea, China	[110]
442	Hypofuran A	Antibacterial and antioxidant	*H.* *koningii* (*T.* *koningii*) PF04	Sponge *Phakellia* *fusca*	Yongxing Island in the South China Sea, China	[77]
443	Hypofuran B		*H.* *koningii* (*T.* *koningii*) PF04	Sponge *Phakellia* *fusca*	Yongxing Island in the South China Sea, China	[77]
444	Hypocrol A	Antibacterial and antioxidant	*H.* *koningii* (*T.* *koningii*) PF04	Sponge *Phakellia* *fusca*	Yongxing Island in the South China Sea, China	[129]
445	Gliocladinin D		*T.* *reesei* HN-2016-018	Unidentified sponge	South China Sea, China	[130]

## Structure and occurrence

### Terpenes

A total of 165 new terpenes (**1**–**165**, Table 1) were isolated and identified from 10 marine-derived *Trichoderma* species, including *T.* *asperelloides* (18 compounds), *T.* *asperellum* (47), *T.* *atroviride* (2), *T.* *brevicompactum* (20), *T.* *citrinoviride* (4), *T.* *erinaceum* (5), *T.* *hamatum* (3), *T.* *harzianum* (27), *T.* *longibrachiatum* (4), and *T.* *virens* (19), and five unidentified strains (16) [25–65]. These terpenes can be classified to monoterpenes with a menthane skeleton, sesquiterpenes with cyclonerane, bisabolane, trichothecane, carotane, cadinane, acorane, cuparane, farnesane, synderane, pupukeanane, and harzianoic acid skeletons, and diterpenes with harziane, proharziane, wickerane, citrinovirin, fusicoccane, cleistanthane, and sordaricin skeletons. The above 19 basic scaffolds along with the degraded and substituted ones demonstrate the high structural diversity of terpenes from marine-derived *Trichoderma*.

**Fig. 6:**
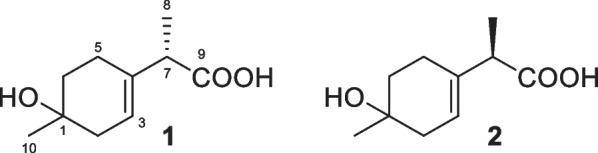
Monoterpenes from marine-derived *Trichoderma*

Monoterpenes have seldom been discovered from *Trichoderma* species, including marine-derived ones. Monoterpenes **1** and **2** (Fig. 6) were obtained from the alga-endophytic *T.* *asperellum* and represent the only two menthane derivatives from this genus [25]. These two compounds with only two chiral centers were identified as mutual epimers by the similar but different NMR data, because those of enantiomers are the same as each other. Their absolute configurations at C-7 were assigned to be *S* and *R*, respectively, by quantum chemical calculations of electronic circular dichroism (ECD) spectra. Based on the epimeric relationship between **1** and **2**, these two metabolites were proposed to have the same absolute configuration at C-1, but it was not determined spectroscopically. As a chlorinated analog of them, 3-chloromenthan-1,2,7-triol had also been isolated from a fungus, *Tryblidiopycnis* sp. of mangrove origin [131]. Based on previous reports, monoterpenes were rarely encountered not only in *Trichoderma* species but also in other marine and even terrestrial-derived fungi [132].

**Fig. 7:**
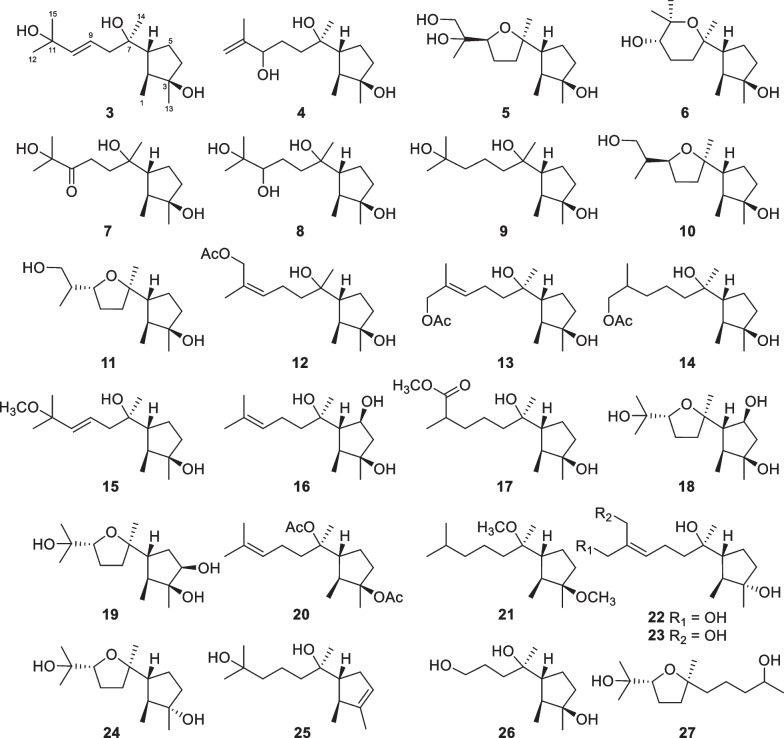
Cyclonerane sesquiterpenes and catabolites from marine-derived *Trichoderma*

**Fig. 8:**
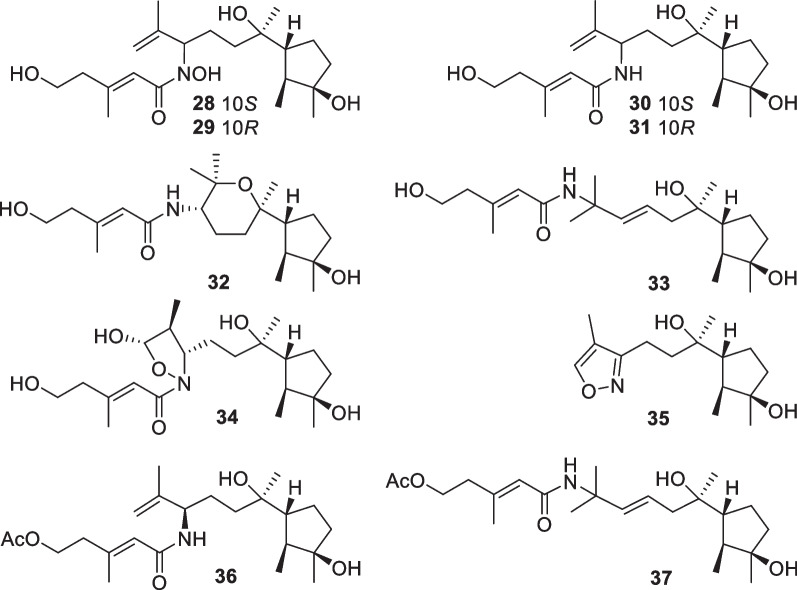
Nitrogenous cyclonerane sesquiterpenes from marine-derived *Trichoderm*

Sesquiterpenes with 126 new members (**3**–**128**, Table 1) make up the largest group of terpenes from marine-derived *Trichoderma*, and they comprise 11 basic carbon skeletons [26–52]. A common cyclonerane skeleton is present in 23 isolates (**3**–**25**, Fig. 7), obtained from *T.* *asperellum* [26–28], *T.* *harzianum* [29, 30], *T.* *hamatum* [31], *T.* *erinaceum* [32], *T.* *citrinoviride* [33], and *T.* *asperelloides* [34]. This scaffold is characterized by the dimethylated cyclopentane ring attached by a 1,6-dimthylhexanyl side chain. Oxidation, reduction, cyclization, and substitution construct their diverse structures. All of them feature an oxygen atom bonded to C-7. The hydroxy group at C-3 of **22**–**24** possesses an opposite orientation, and the double bond in the five-membered ring of **25** also renders this molecule unique [33, 34]. Two isolates (**26** and **27**, Fig. 7) from *T.* *asperellum* and *T.* *asperelloides*, respectively, have degraded cyclonerane frameworks, with the degradation happening at the side chain or the ring unit [28, 34]. In addition, 10 nitrogenous cyclonerane derivatives (**28**–**37**, Fig. 8) were obtained from *T.* *asperellum* [27, 35]. Each of them contains a nitrogen-bearing substitute, and the highlight is the presence of an isoxazole ring in **35** [27]. Cyclonerins A (**28**) and B (**29**) harbor a hydroxamic acid unit, that can chelate ferric ion, and represent the first two fungal hydroxamic acids with a terpene-derived scaffold [27]. The identification of these compounds was performed through various spectroscopic methods. Quantum chemical calculations were used to aid the assignments of relative configurations for **22** and **26** and absolute configurations for **22**, **28**–**32**, and **34**, and X-ray diffraction was employed to determine the absolute configuration of **6** [27, 28, 33]. Although the relative configuration at C-7 of **7**–**9** and **12** was not given in literature [28], it should be the same as that **26** based on biogenetic considerations. Cyclonerane sesquiterpenes, especially the known cyclonerodiol, can be produced by many species of *Trichoderma* and other fungal genera, such as *Trichothesium*, *Fusarium*, and *Epichloe* [33], but they have rarely been detected in plants and animals. It is worth mentioning that marine-derived *Trichoderma* strains have contributed more diverse cycloneranes than terrestrial-derived ones so far [20, 22].

**Fig. 9:**
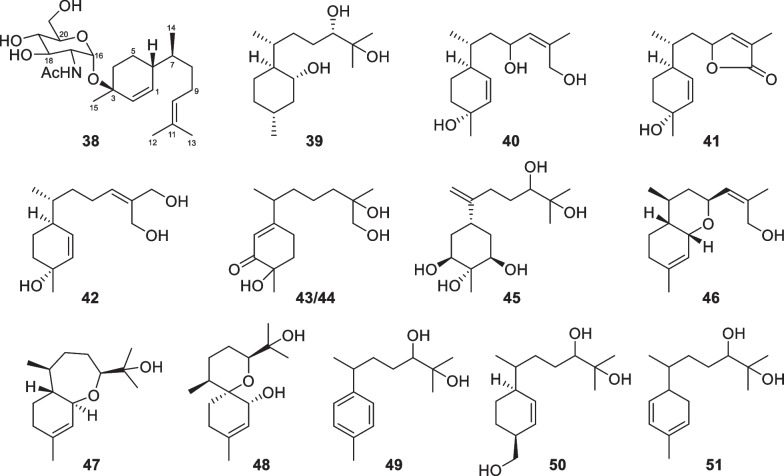
Bisabolane sesquiterpenes from marine-derived *Trichoderma*

**Fig. 10:**
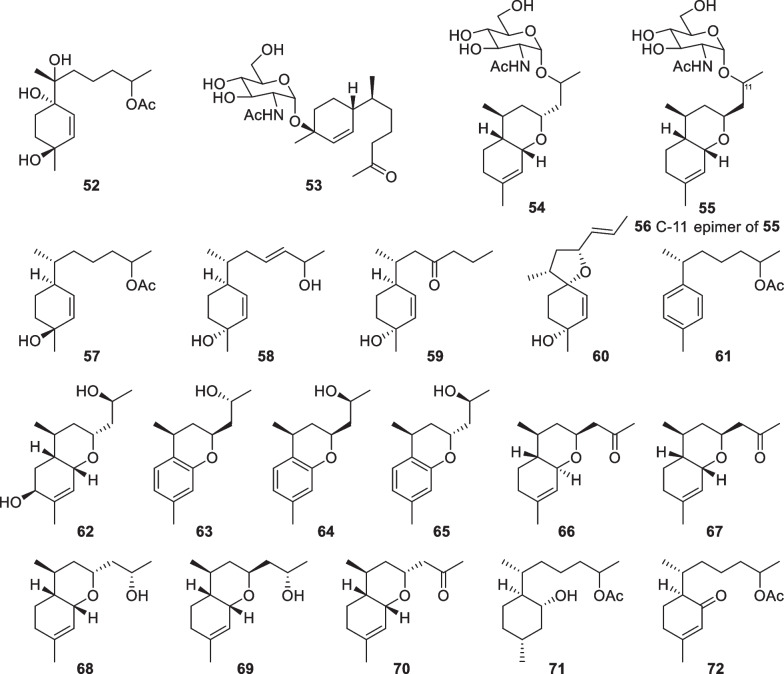
Norbisabolane sesquiterpenes from marine-derived *Trichoderma*

Bisabolane sesquiterpenes have been known as metabolites of plants, animals, and microbes for a long time, but the first discovery from *Trichoderma* just happened in 2011 [133, 134]. Bisabolane derivatives from marine-derived *Trichoderma* also exhibit high structural diversity. 14 members (**38**–**51**, Fig. 9) with an untouched bisabolane skeleton were isolated from four *Trichoderma* species, including *T.* *asperellum* [25, 26, 36], *T.* *brevicompactum* [37], *T.* *asperelloides* [34], and *T.* *erinaceum* [32]. Among them, trichaspside A (**38**) represents the first natural bisabolane aminoglycoside, and all the others possess oxygenated side chain termini. Meanwhile, 21 norbisabolane sesquiterpenes (**52**–**72**, Fig. 10), with four (**53**–**56**) containing an aminoglycoside moiety, were discovered from *T.* *asperellum* [25, 26, 36, 38], *T.* *atroviride* [39], and *T.* *asperelloides* [34]. All the norbisabolanes are possibly produced by elimination of a terminal methyl group from the side chain moieties of their precursors, and the majority of them bear an oxygen atom at C-11. It is regretted that absolute configurations for most of the bisabolane and norbisabolane derivatives remain unsolved because of lacking ECD signals and perfect crystals. However, trichobisabolin Z (**72**) has a Cotton effect at 328 nm due to the presence of an α,β-unsaturated carbonyl group, which enabled the assignment of the absolute configuration at C-6 by quantum chemical calculations [34]. An acidic hydrolysis reaction was performed during the absolute configuration establishment of **38** [26]. Trichodermaerin A (**51**) with no optical activity was deduced to be a racemic mixture, but the separation failed via various chiral HPLC columns [32]. In general, the high populations of norbisabolane sesquiterpenes and aminoglycosides may be characteristic of marine-derived *Trichoderma*.

**Fig. 11:**
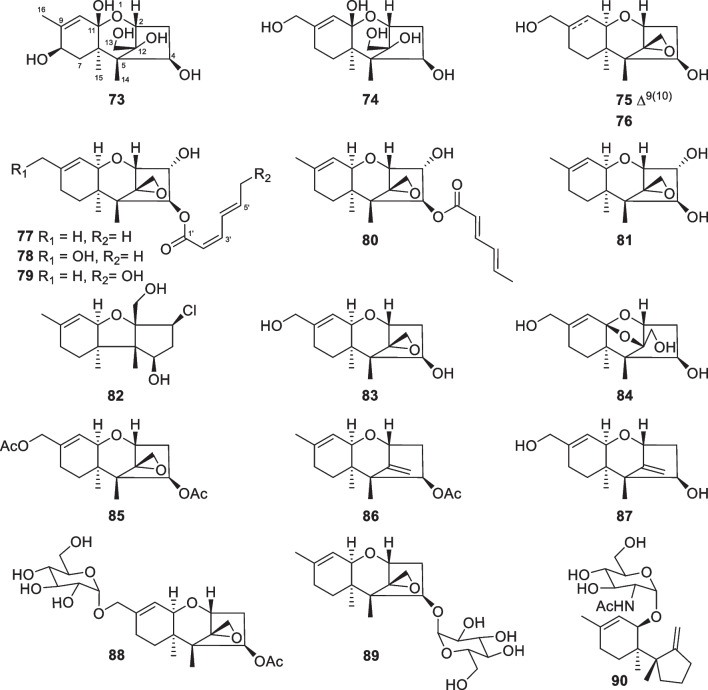
Trichothecane sesquiterpenes from marine-derived *Trichoderma*

As secondary metabolites of some *Trichoderma* and *Fusarium* species, trichothecane sesquiterpenes have been regarded as a class of mycotoxins for animals and humans [20]. Besides the known trichodermin, 18 trichothecane derivatives (**73**–**90**, Fig. 11) were isolated from marine-derived *Trichoderma* species, including *T.* *brevicompactum* and *T.* *hamatum* as well as an unidentified strain [31, 40–43]. Similar to the known trichothecane sesquiterpenes [135], the majority of these new isolates possess a 2,11-epoxy unit. However, trichodermol chlorohydrin (**82**) and trichodermarin N (**90**) are exceptions, with the former being the first natural halogenated trichothecane derivative. Both 2,11-epoxy and 11,12-epoxy units are present in trichodermarin H (**84**), of which the absolute configuration along with that of trichodermarin G (**83**) was assigned by X-ray crystallography. The absolute configurations of **77**–**80** were ascertained by analysis of their ECD data aided by quantum chemical calculations, while that of **81** was determined by agreement of its specific optical rotation with the hydrolysate of **77**. It is interesting that glycosides are also not absent in this class of metabolites. Three members (**88**–**90**) were identified to possess sugar moieties, with **90** being the first glucosamine-coupled trichothecane. It is worth noting that the production of trichothecane toxins may decrease the application of their producers, though some strains display excellent antifungal effects [136].

**Fig. 12:**
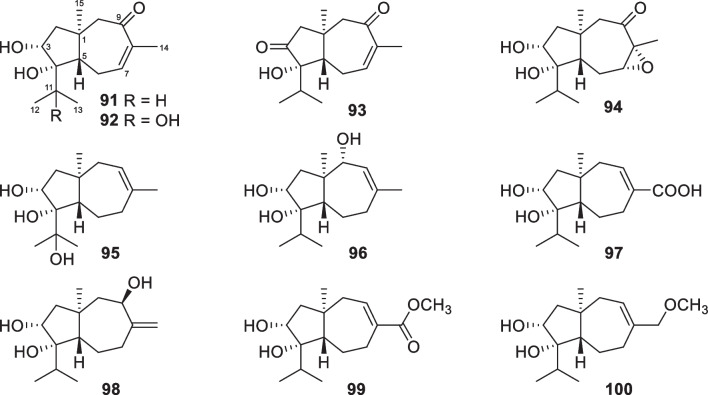
Carotane sesquiterpenes from marine-derived *Trichoderma*

Carotane, also designated daucane, sesquiterpenes with 10 new members (**91**–**100**, Fig. 12) were purified from two strains of the marine algicolous *T.* *virens* [44, 45]. Among *Trichoderma* species, *T.* *virens* is the main producer of carotane sesquiterpenes, but their origin is not confined to only this species. A soil-borne *T.* *crassum* strain was also reported to yield carotanes [137]. All these 10 isolates are oxygenated at C-3 and C-4, and the peculiarity is a carbonyl group, rather than a hydroxy group, at C-3 of trichocarotin C (**93**). The absolute configuration of trichocarotin A (**91**) was determined by analysis of X-ray crystallographic data, while those of trichocarotin B (**92**) and 14-*O*-methyltrichocarotin G (**99**) were established by ECD spectra. Although other filamentous fungi, such as *Byssochlamys*, *Penicillium*, and *Aspergillus* species, also produce carotane sesquiterpenes, the oxidation at C-3 and C-4 are uncommon [138]. Thus, the oxygenated carotanes at both C-3 and C-4 have chemotaxonomic significance for their *Trichoderma* producers.

**Fig. 13:**
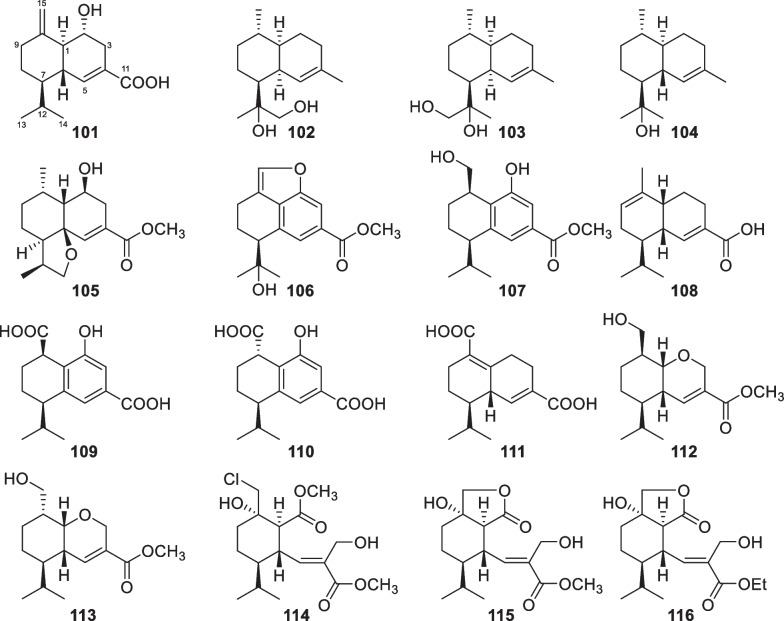
Cadinane sesquiterpenes and catabolites from marine-derived *Trichoderma*

Besides carotanes, cadinane sesquiterpenes also exist in *T.* *virens* [44, 45]. However, this class of sesquiterpenes (**101**–**116**, Fig. 13) are distributed in a broader spectrum of other *Trichoderma* species, such as *T.* *asperelloides* [34], *T.* *asperellum* [46], *T.* *harzianum* [48], and an unidentified strain [47]. The first untouched cadinane sesquiterpene trichocadinin A (**101**) from *Trichoderma* was reported in 2018, even if a 2,3-seco derivative, named heptelidic acid, was found from *T.* *viride* in 1980 [20]. The 2,3-seco cadinane sesquiterpenes (**114**–**116**) were also obtained from two other *Trichoderma* species of marine origin, with halogenation being present in **114**. Moreover, trichocadinins I (**112**) and J (**113**) possess a 2-norcadinane framework. Except for **102–104**, these cadinane derivatives feature a carboxyl group at C-11. Relying on the presence of a conjugated carboxyl group, the absolute configurations of **101** and **105**–**115** were established by ECD spectra. Additionally, the absolute configuration of **104** was determined by X-ray diffraction. Although cadinane sesquiterpenes have occurred frequently in plants and fungi, the ring-opening derivatives are distributed narrowly in nature [139].

**Fig. 14:**
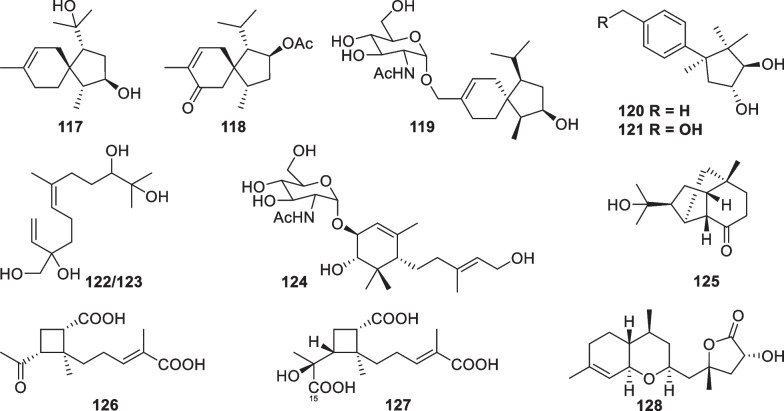
Other sesquiterpenes from marine-derived *Trichoderma*

Other 12 sesquiterpenes (Fig. 14) with acorane (**117**–**119**) [30, 37, 49], cuparane (**120** and **121**) [43], farnesane (**122** and **123**) [37], synderane (**124**) [50], norpupukeanane (**125**) [51], harzianoic acid (**126** and **127**) [52], and ethylated bisabolane (**128**) [26] frameworks were discovered from *T.* *harzianum*, *T.* *brevicompactum*, *T.* *asperellum*, *T.* *longibrachiatum*, and an unidentified strain. 8-Acoren-3,11-diol (**117**), trichoacorin A (**118**), and trichoacorside A (**119**) are the only three spiro-fused sesquiterpenes characterized from marine-derived *Trichoderma*, with **119** being the first acorane aminoglycoside. Trichocuparins A (**120**) and B (**121**) represent the first two *Trichoderma*-derived cuparane derivatives, and the absolute configuration of the former was determined by analysis of X-ray crystallographic data. This carbon skeleton harbors the same ring system as trichothecane, but one of the four methyl groups resides at a different position. Trichonerolins A/B (**122**/**123**), trichodermoside (**124**), and trichodermene A (**125**) are also firstly occurring structural types in *Trichoderma* metabolites, especially an aminosugar unit in **124** and a complicated ring system in **125**. There are two chiral centers in **122** and **123**, of which the chemical shift deviations undoubtedly arise from their epimeric relationship. Harzianoic acid B (**127**) features a new natural scaffold with a cyclobutane nucleus, and harzianoic acid A (**126**) is its 15-nor derivative. In addition, the ethylated bisabolane skeleton of trichaspin (**128**) is unprecedented, too. Most of these skeletons have been discovered in only one or two species, their universality in marine-derived *Trichoderma* needs to be further explored.

**Fig. 15:**
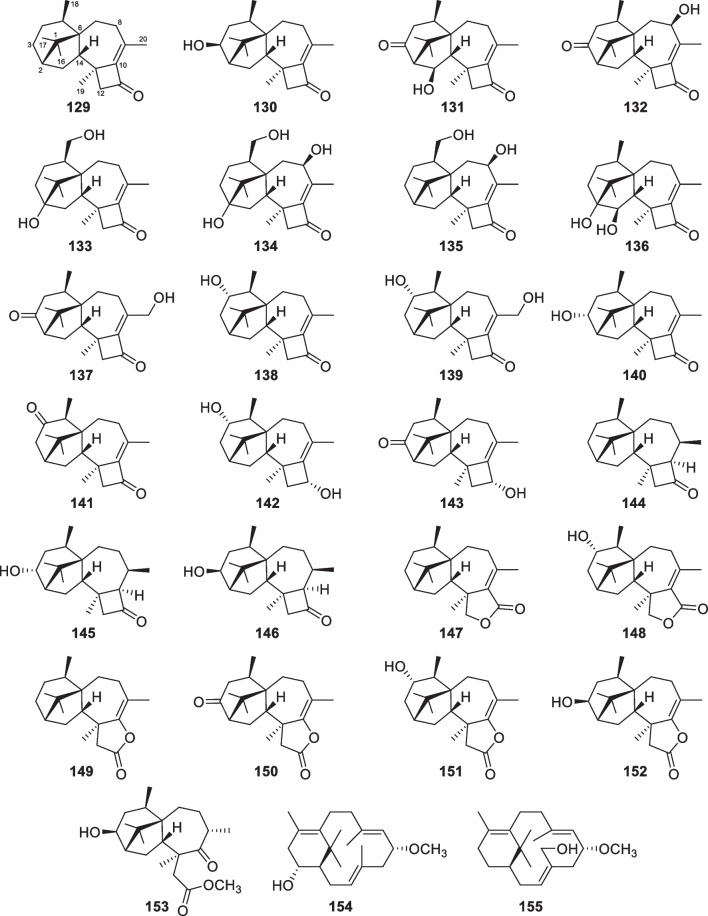
Harziane, secoharziane, and proharziane diterpenes from marine-derived *Trichoderma*

The number of new diterpenes with seven basic carbon skeletons from marine-derived *Trichoderma* amounts to 37 (**129**–**165**, Table 1) [26, 30, 32, 46, 53–65]. Of those, 25 members (**129**–**153**, Fig. 15) are harziane derivatives, that were isolated from *T.* *asperellum* [26, 46], *T.* *longibrachiatum* [53, 54], *T.* *harzianum* [30, 56, 57], *T.* *asperelloides* [59], *T.* *erinaceum* [32, 60], and two unidentified strains [55, 58]. The common harziane skeleton harbors a fused cyclobutane, cyclopentane, cyclohexane, and cycloheptane ring system, that is unique in nature. Harzianone (**129**) was reported as the second harziane diterpene in 2012, 20 years later than the discovery of harziandione with a second carbonyl group at C-3. The absolute configuration of **129** was ascertained by quantum chemical calculations of ECD data, and that of harziandione was confirmed by simulation of the specific optical rotation at the same time [53]. Other isolates with this rigid scaffold have different oxidation degrees and positions, and the double bond at C-9 is hydrogenated in **144**–**146** in particular. The first total synthesis of a harziane diterpene was reported in 2020, with highly diastereocontrolled construction of the cyclobutane ring via enyne cycloisomerization being a key step [140]. This work also resulted in configurational revision at C-9 of the target harzian-9-ol, isolated from the tree-associated *T.* *atroviridae*. Harzianelactone (**147**) and trichodermaerin (**150**) represent the first 11,12-lactone and 10,11-lactone, respectively, that are possibly produced by catalysis of the Baeyer–Villiger monooxygenase [58, 60]. X-ray diffraction with Mo Kα radiation secured the relative configuration of **150**, that was isolated from *T.* *asperellum* later [141]. Lactonation seemingly happens only in the four-membered ring, and a subsequent reaction may yield ring-opening products, such as **153** [59]. In addition, two proharziane diterpenes (**154** and **155**) with a 14-membered macroring being inlaid with a cyclohexane unit were identified from *T.* *asperelloides* and *T.* *harzianum* [30, 59], and they seem intermediates during the biosynthesis of corresponding harzianes [142]. The absolute configurations of all these 27 isolates were confirmed by chiral techniques. As the assignment of **129**, ECD analysis played an important role in determining the absolute configurations of most isolates. X-ray diffraction was also used to determine the absolute configurations of several molecules, including **131**, **132**, **140**, and **142**. The distribution of harziane, secoharziane, and proharziane diterpenes is not confined to only the above *Trichoderma* species, and they rarely occur in other fungal genera. Thus, these diterpenes with tetracyclic and bicyclic scaffolds are promising to be regarded as biomarkers for *Trichoderma* fungi.

**Fig. 16:**
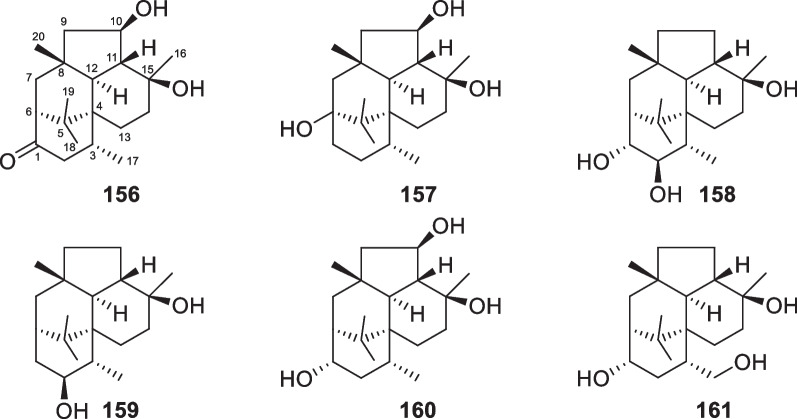
Wickerane diterpenes from marine-derived *Trichoderma*

Wickerane diterpenes look like exclusive metabolites of *Trichoderma*, six members (**156**–**161**, Fig. 16) were discovered from sponge-derived *T.* *harzianum* [61, 62]. Their distribution is not as broad as that of harziane derivatives. *T.* *atroviride* of soil or plant origin contributed the first two wickerane diterpenes, wickerols A and B (also named trichodermanin A), and their biogenetic route was predicted with ^13^C-labeled acetate [143, 144]. In the pathway, the verticillyl cation is the same as the intermediate in the biosynthesis of harziane diterpenes [142, 143]. The complicated tetracyclic scaffold and its relative configuration were guaranteed by X-ray crystallographic analysis [144]. A stereocontrolled synthesis from commercial sitolactone led to the assignment of absolute configurations and the revision of the original specific optical rotation sign of wickerol A [145]. Compared to wickerols A and B, trichodermanins C–H (**156**–**161**) feature high degrees of oxidation. The lack of any olefinic and aromatic unit is also characteristic of these diterpenes. The absolute configurations of **156**, **157**, and **159** were determined by application of the modified Mosher’s method, while that of **158** was established by the negative Cotton effect at 258 nm in the ECD spectrum of its dibenzoate. In view of the narrow distribution spectrum, this class of diterpenes may also be potential biomarkers for several *Trichoderma* species.

**Fig. 17:**
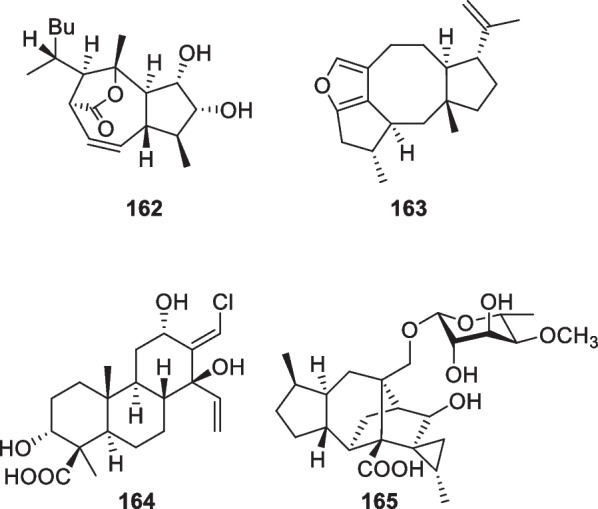
Other diterpenes from marine-derived *Trichoderma*

Besides the above harziane and wickerane, there are also four diterpene skeletons (Fig. 17) occurring in marine-derived *Trichoderma*. Citrinovirin (**162**) with a new norditerpene skeleton and trichocitrin (**163**) with a fusicoccane skeleton were isolated from *T.* *citrinoviride* [63, 64]. Harzianolic acid A (**164**) with a cleistanthane skeleton and trichosordarin A (**165**) with a 15-nor transformed sordaricin skeleton were obtained from *T.* *harzianum* [56, 65]. The latter three metabolites (**163**–**165**) are firstly occurring structural types in this genus, and the furan ring in **163**, the chlorine atom in **164**, and the deoxymonosaccharide in **165** greatly enhance their novelty.

### Steroids

**Fig. 18:**
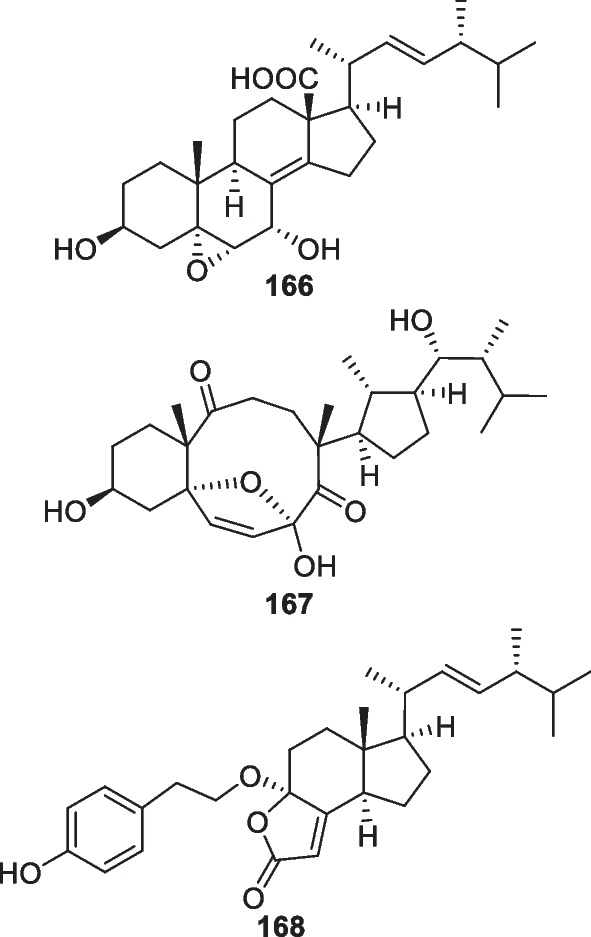
Ergosterol derivatives from marine-derived *Trichoderma*

Compared to the high number of terpenes, only three new steroidal metabolites (**166**–**168**, Fig. 18) with ergostane and its transformed and degraded skeletons were discovered from marine-derived *Trichoderma* species, including *T.* *brevicompactum* [37], *T.* *asperellum* [66], and *T.* *atroviride* [39]. It is worth mentioning that all these isolates feature high degrees of oxidation, that even results in the broken ergostane scaffolds in **167** and **168**. Considering the structural complexity, the assignments of absolute configurations of **167** and **168** were completed by both ECD and X-ray crystallographic analyses. Unfortunately, no steroidal metabolites of the viridin series have been found in marine-derived *Trichoderma* [20].

### Polyketides

Despite the lower number of new polyketides (**169**–**295**) than that of terpenes, they have more prolific sources. Totally, 14 marine-derived *Trichoderma* species, including *T.* *asperellum* (2 compounds), *T.* *atroviride* (6), *T.* *aureoviride* (2), *T.* *citrinoviride* (3), *T.* *erinaceum* (6), *T.* *harzianum* (27), *T.* *koningii* (9), *T.* *longibrachiatum* (3), *T.* *reesei* (32), *T.* *saturnisporum* (8), *T.* *virens* (3), *T.* *viride* (1), *H.* *lixii* (1), and *H.* *vinosa* (2), and nine unidentified strains (22) have the ability to produce polyketides [24, 25, 32, 33, 39, 49, 67–106]. These metabolites can be classified into cyclopentenone, sorbicillinoid, koninginin, decalin, xanthone, anthraquinone, naphthopyrone, and other acyclic and cyclic categories, with several novel skeletons being present.

**Fig. 19:**
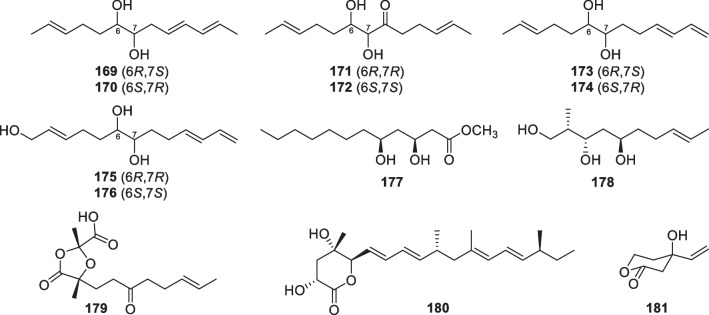
Acyclic polyketides and lactones from marine-derived *Trichoderma*

As shown in Fig. 19, four pairs of C_13_ lipids named harzianumols A-H (**169**–**176**) were isolated from the sponge-associated *T.* *harzianum* [67]. Each pair of them occurred as an inseparable enantiomeric mixture, and their absolute configurations were identified through the modified Mosher’s method. As a possible derivative of lauric acid, oxylipin **177** was discovered from the alga-derived *T.* *atroviride* [39]. Additionally, two methyl-branched lipids (**178** and **179**) with the same backbone were identified from *T.* *citrinoviride*, and the latter contains a unique 1,3-dioxolane nucleus [68, 69]. Nafuredin C (**180**) with a δ-lactone unit was obtained from the mangrove-endophytic *T.* *harzianum*, occurring as the third member of this structural family [70]. An algicolous *T.* *citrinoviride* strain can also yield a member of this type [64], but the producers of nafuredins are not only *Trichoderma* but also *Aspergillus*, *Penicillium*, and *Talaromyces* [146, 147]. *T.* *harzianum* of sponge origin gave harzialactone B (**181**), of which the δ-lactone moiety seems formed through the Baeyer–Villiger oxidation of a cyclopentenone precursor [71].

**Fig. 20:**
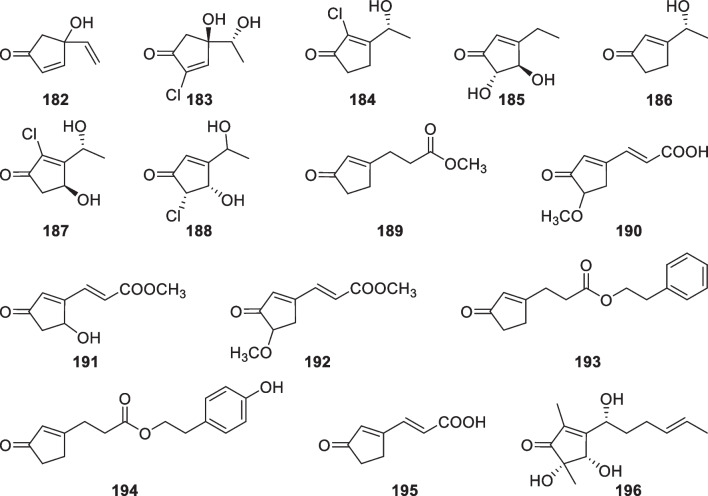
Cyclopentenone derivatives from marine-derived *Trichoderma*

Cyclopentenone derivatives with 15 members (**182**–**196**, Fig. 20) were discovered from *T.* *harzianum* [71, 72], *T.* *asperellum* [25], *T.* *koningii* [74, 77], *T.* *atroviride* [75, 76], and two unidentified strains [73, 78]. This class of metabolites are structurally simple, and all of them contain an α,β-unsaturated carbonyl group in the five-membered ring. Seven (**182**–**188**) of the isolates possess only seven carbon atoms, with one olefinic carbon adjacent to the carbonyl group tending to be chlorinated. One more carbon atom is present the nucleic moieties of **189**–**195**, which exist as esters or acids without a chlorine atom at α position of the carbonyl group. The structure of **196** differs from those of the others by the presence of a long side chain and two methyl groups on the cyclopentenone nucleus. The configurational assignments of **182**–**184** were achieved by total syntheses, with the former one being speculated as scalemic enantiomers by consideration of its smaller specific rotation than that of the synthesized one [72]. On the other hand, the conjugated carbonyl group facilitates the stereochemical determination of some chiral members, such as **186**, **187**, and **196**, by ECD data [25, 78]. In spite of the similarity between cyclopentenones and isonitriles of *Trichoderma* origin, the former should be synthesized through polyketide synthases, but the latter were deduced to be derived from tyrosine [148].

**Fig. 21:**
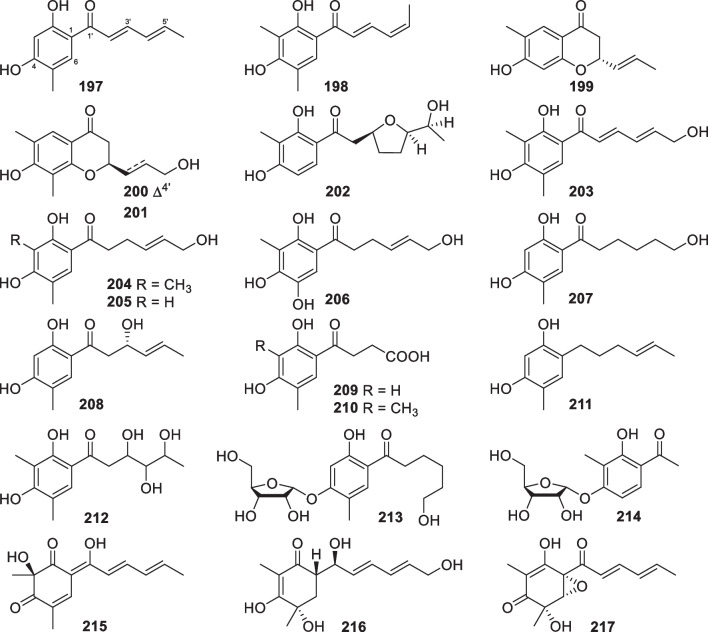
Monomeric sorbicillinoids from marine-derived *Trichoderma*

**Fig. 22:**
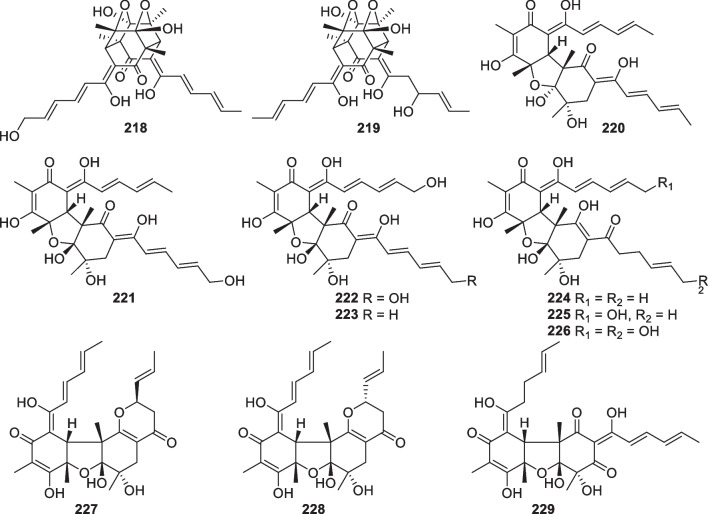
Dimeric sorbicillinoids from marine-derived *Trichoderma*

**Fig. 23:**
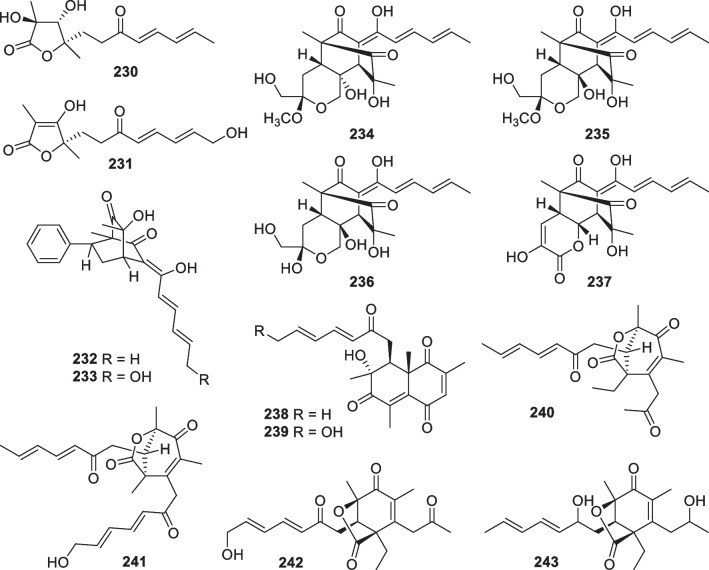
Other sorbicillinoid derivatives from marine-derived *Trichoderma*

As the largest group of polyketides from marine-derived *Trichoderma*, sorbicillinoids and their derivatives consist of 47 new members. Monomeric sorbicillinoids (**197**–**217**, Fig. 21) from *T.* *reesei* [81, 82], *T.* *saturnisporum* [84], *T.* *longibrachiatum* [49, 85], and three unidentified strains [79, 80, 83] are the most primitive representatives, and the majority of them are constructed with a phenyl core substituted by a sorbyl-derived acyclic or epoxy side chain and one or two methyl groups. Methylation happens at C-3 and/or C-5 before cyclization of the hexaketide chain by the Claisen reaction [149], and hydroxylation commonly occurs at C-2 and/or C-4. However, the C-5 position of **206** is also bonded to a hydroxy group, rather than a methyl group. **202**, **209**, **210**, and **214** harbor nonstandard sorbyl chains, possibly arising from the length change in original polyketide chains or oxidation scission of C_6_ units. Based on biogenetic considerations, saturnispol E (**216**) and epoxysorbicillinol (**217**) with a cyclohexene core are possible deoxy and epoxy derivatives of the tautomeric intermediates of sorbicillin, while (-)-trichodermatone (**215**) is a regioisomer of sorbicillinol [149]. In addition, 12 dimeric sorbicillinoids (**218**–**229**, Fig. 22), also called bisorbicillinoids, were obtained from *T.* *reesei* [81, 82, 86], *T.* *saturnisporum* [84], and one unidentified strain [79]. All the isolates are heterogeneous dimers, and their skeletal dimerization happens between C-3 of one monomer and C-6 of the other. Their structures appear more complicated, especially the unique cage-like core in **218** and **219**. These dimers were proposed to be constructed through intermolecular single or double Michael reaction/ketalization, with sorbicillinol or its analogs serving as key intermediates [150]. Apart from the above monomers and dimers, there are also 14 other sorbicillinoid derivatives (**230**–**243**, Fig. 23) from marine-derived *T.* *saturnisporum* [84], *T.* *reesei* [82, 86], and two unidentified strains [83, 87]. 2,3-Dihydro 2-hydroxy vertinolide (**230**) and saturnispol F (**231**) belong to the vertinolide subfamily, with the lactone unit being probably formed via intramolecular esterification and ring cleavage sequences. Saturnispols C (**232**) and D (**233**) feature a bicyclo[2.2.2]octanedione motif that arises from a Diels–Alder [4 + 2] cycloaddition between the corresponding sorbicillinoid monomer and phenylethylene. Based on the same mechanism, the formation of each trichodermanones A-D (**234**–**237**) appears rationalized by reaction between sorbicillinol and a dienophile. The last six members (**238**–**243**) differ greatly from the others, due to the presence of a methylene group between the sorbyl chains and the six-membered ring. Moreover, a naphthalene ring is present in **238** and **239**, and a bicycle [3.2.1] lactone unit appears in **240**–**243**. During the identification of chiral isolates, ECD spectra were widely used to confirm their absolute configurations, and X-ray diffraction was also used for several members, such as **200**, **202**, **206**, and **214**. A total synthesis of (±)-epoxysorbicillinol was fulfilled from commercial diethyl methylmalonate in 13 steps, simultaneously permitting epoxidation and avoiding aromatization [151]. A conversion from sorbicillin to (±)-epoxysorbicillinol was also achieved via the formation of a *p*-quinol intermediate by an oxidative dearomatization [152]. To date, the number of sorbicillinoid members exceeds 130, and they have been discovered from no less than 10 genera of ascomycetes [86, 153]. It is interesting that all the known marine-derived *Trichoderma* species that produce sorbicillinoids belong to the Longibrachiatum clade [154].

**Fig. 24:**
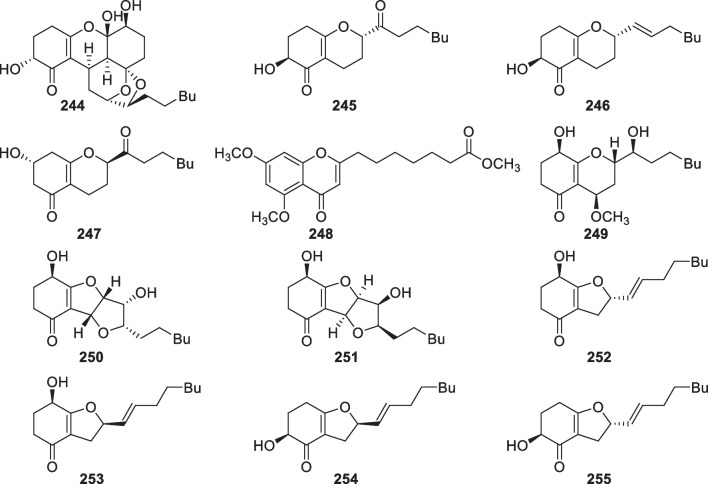
Koninginins and their derivatives from marine-derived *Trichoderma*

As a family of octaketides, koninginins with a narrow distribution are also the representative metabolites from *Trichoderma*. To date, 12 new members (**244**–**255**, Fig. 24) have been isolated from marine-derived *T.* *reesei* [89], *T.* *koningii* [91], *H.* *lixii* [90], and one unidentified strain [92]. The first koninginin, named koninginin A, was obtained from *T.* *koningii* in 1989 [20], and it defined the typical koninginin skeleton that contains a benzopyran nucleus and a heptyl side chain. This skeleton is present in **245**–**249**, especially a highly unsaturated chromone motif in **248**. In **244**, a second cyclohexene ring is incorporated into this basic structure, forming an unprecedented pentacyclic framework with a ketalic and a hemiketalic carbon. Starting from L-tartaric acid, a synthetic route for this complicated molecule, that harbors eight chiral carbons, was developed in a stereocontrolled manner [155]. Instead of the pyran ring, one or two furan rings exist in **250**–**255**. The former two are unprecedented due to the presence of a ditetrahydrofuran-bearing tricyclic skeleton. Although ECD spectra were applied to confirming the absolute configurations for most of the isolates [89, 91, 92], the relative configurations between the chiral carbon in cyclohexene ring and that in pyran or furan ring failed to be given in some structures due to the lack of any NOE correlation. Chemical syntheses suggested that epimeric pairs possess very similar spectroscopic data, which also make it difficult to distinguish their relative configurations by comparison of spectroscopic data [156].

**Fig. 25:**
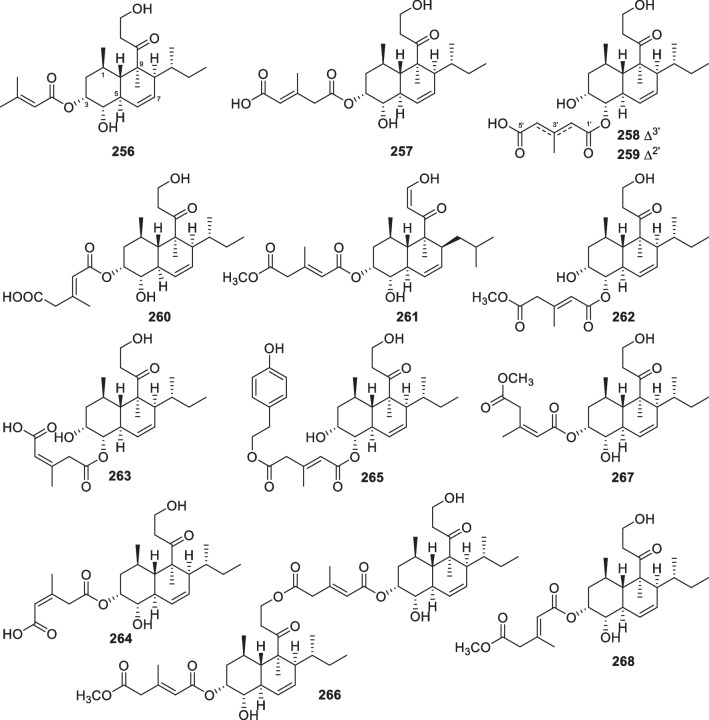
Decalin derivatives from marine-derived *Trichoderma*

Decalin derivatives, such as trichosetin and compactin, were previously detected in several *Trichoderma* species [20]. Marine-derived *T.* *harzianum* along with an unidentified strain gave another type of decalin derivatives (**256**–**268**, Fig. 25), with a C_3_ and a C_4_ side chain as well as two methyl groups [24, 93–98]. The bicyclic nucleus is a *trans*-fused ring system, with a double bond at C-6. Acylation of hydroxy groups on the decalin core and the C_3_ side chain contributes to the structural diversity of this octaketide type, especially the formation of the dimer **266**. Except for trichoharzianin (**256**), the acyl groups are confined to (*Z*)- and (*E*)-3-methylpent-2-enedioic acid (3-methylglutaconic acid) and their monoesters, which are presumed to arise from mevalonic acid [24]. A tyrosol residue that can be converted from tyrosine exists in **265** [157], similar to **168** and **194**. The absolute configurations of most members were assigned by chemical conversion, including alkali-hydrolysis to yield triols or further acylation to furnish tribenzoates. Dimolybdenum tetraacetate [Mo_2_(OAc)_4_] was used to form complexes with the *cis* vic-diol group in hydrolysates of **257**–**259**, and analyses of their ECD spectra resulted in determination of the absolute configurations. Analogs with the same or similar alkylated decalin skeletons were also identified from other fungal species of the genera *Eupenicillium*, *Fusarium*, *Geomyces*, *Phoma*, *Spopormiella*, and *Stemphylium* [95, 96], but the 3-methylglutaconate units were rarely encountered therein.

**Fig. 26:**
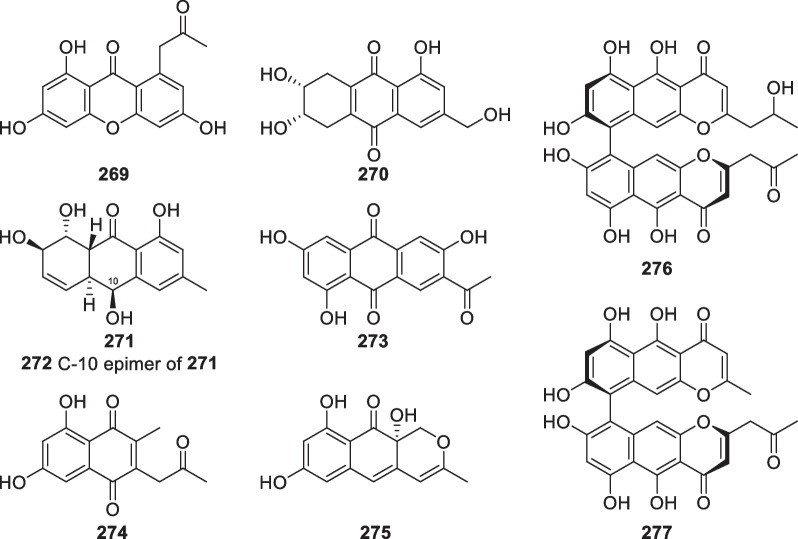
Xanthone, anthraquinone, and naphthopyrone derivatives from marine-derived *Trichoderma*

Xanthone, anthraquinone, and naphthopyrone derivatives have been discovered from multifarious plants and/or fungi [158, 159], but their distribution in *Trichoderma* is not much attractive. Only nine new members (**269**–**277**, Fig. 26) were obtained from marine-derived *Trichoderma*, including *T.* *aureoviride* [99], *T.* *harzianum* [100], *H.* *vinosa* [102], and one unidentified strain [101]. Trichodermaxanthone (**269**) contains a typical xanthone skeleton, which rarely occurs in the metabolites of this genus. Octaketides **270**–**273** possess a basic anthraquinone scaffold, but one of the two carbonyl functionalities is reduced to a hydroxy group in epimers **271** and **272**. Considering the similarity between **274** and **275**, they possibly arise from the same heptaketide intermediate. Hypochromins A (**276**) and B (**277**) have a dimeric naphtho-γ-pyrone framework, which are present in a broad spectrum of ascomycetous producers [160]. There is one or more chiral centers in **271**, **272**, and **275**, of which the configurations were determined by interpretation of ECD spectra. A crystallographic analysis further confirmed the absolute configuration of **271**. In addition, **276** and **277** with atropisomerism are axially chiral molecules, and the single bond between the two monomers was assigned as *S*-configuration by the exciton chirality method. These highly unsaturated isolates exhibit yellow or red colors, which may be taken as pigments by their producers.

**Fig. 27:**
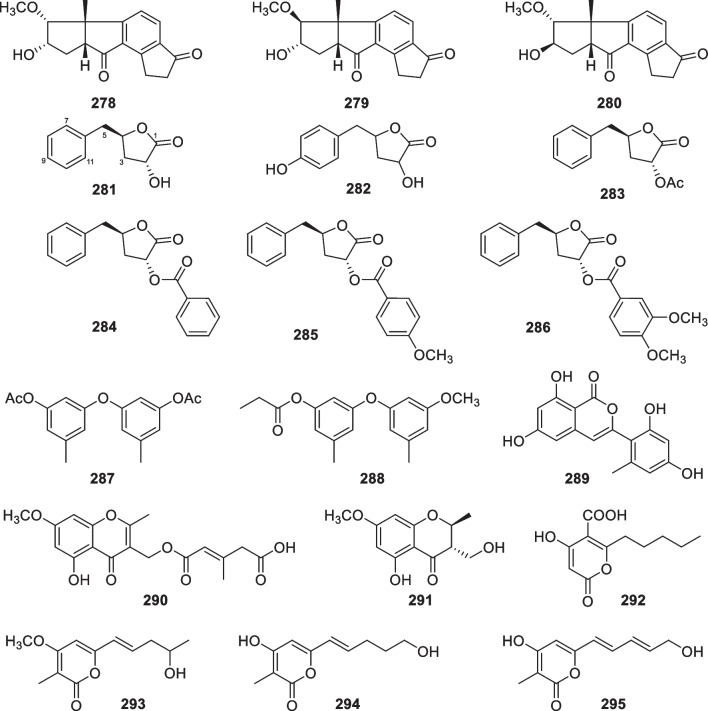
Other aromatic polyketides from marine-derived *Trichoderma*

In addition to the above sorbicillinoid, xanthone, anthraquinone, and naphthopyrone derivatives, a few other aromatic polyketides (**278**–**295**, Fig. 27) with one or two benzene, furan, and/or pyran rings have been isolated from marine-derived *Trochoderma*. Trichorenins A-C (**278**–**280**) from *T.* *virens* harbor a 5/5/6/5-fused ring system, probably arising from the cyclization of an octaketide intermediate [103]. This tetracyclic scaffold had been reported previously, but it is contained in a diterpene with five methyl groups formed through a mevalonate pathway [161]. These two skeletons with the same ring system were guaranteed by single-crystal X-ray diffraction analysis, respectively [103, 161]. The structure of harzialactone A (**281**) with a benzylfuran motif from *T.* *harzianum* is shared in **282**–**286** from *T.* *atroviride* and *T.* *erinaceum* [32, 71, 104, 105]. Except for **282**, these analogous metabolites adopt the same 2*R* and 4*R* configurations. As established by the modified Mosher’s method, the original 2*S* and 4*S* configurations of **281** were corrected by synthesis from monoacetone-D-glucose via a regioselective oxidation [104]. Later on, this compound and its stereo isomers were further synthesized from simple benzene derivatives through several other stereoselectively chemical and enzymatic routes [162–164]. The absolute configurations of **283**–**286** were assigned by comparison of experimental and calculated specific optical rotation data [32]. It is worth mentioning that polyketides of this series have been found not only in *Trichoderma* but also in *Aspergillus* [71]. Two diphenyl ethers, including a symmetrical (**287**) and an asymmetrical derivative (**288**), were identified from *T.* *erinaceum* [32]. This class of metabolites have been detected in various fungal genera, especially in *Aspergillus* and *Penicillium* [165, 166], and they were proposed to be formed through polyketide pathways by analysis of the biosynthetic gene cluster [167]. As a metabolite of *T.* *citrinoviride*, trichophenol A (**289**) is the only 3-phenylisocoumarin member from this genus [33]. Its structure appears similar to flavonoids, and the isocoumarin nucleus is also present in azaphilones of *Trichoderma* origin [20]. A chromone unit exists in **290** and **291**, which were isolated from an unidentified *Trichoderma* strain of hydrothermal vent sediment origin [94]. They are structurally similar to **248**, but their substitution patterns and side chain lengths appear different. Four simple α-pyrone derivatives, named trichoharzianone (**292**), trichopyrone (**293**), and saturnispols G (**294**) and H (**295**), were obtained from *T.* *harzianum*, *T.* *viride*, and *T.* *saturnisporum* [84, 93, 106]. The latter two were postulated to be yielded by elimination of a C_3_ fragment from the precursor sorbicillinol, followed by a lactonization reaction [84].

### Peptides

Comprising 131 new members (**296**–**426**), peptides are the second largest family of metabolites from marine-derived *Trichoderma*. They were identified from seven *Trichoderma* species, including *T.* *asperellum* (42 compounds), *T.* *atroviride* (41), *T.* *harzianum* (1), *T.* *longibrachiatum* (32), *T.* *orientale* (1), *T.* *reesei* (1), *T.* *virens* (4), and two unidentified strains (9) [25, 35, 41, 70, 107–124]. Their structural types involve cyclic and linear peptides, with the latter being composed of dipeptides, aminolipopeptides, peptaibols, and other peptaibiotics.

**Fig. 28:**
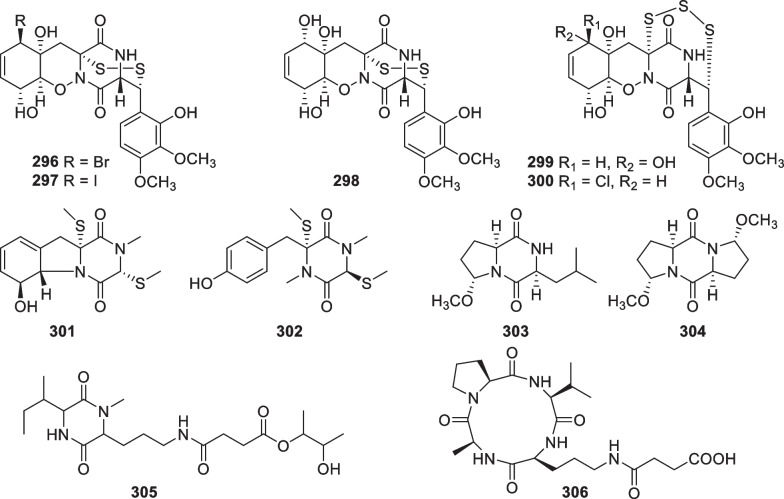
Cyclopeptides including diketopiperazines from marine-derived *Trichoderma*

Cyclopeptides contain 10 diketopiperazines and one cyclotetrapeptide (**296**–**306**, Fig. 28), discovered from *T.* *asperellum* [25, 35], *T.* *virens* [109], *T.* *atroviride* [110], *T.* *reesei* [111], and one unidentified strain [41, 107, 108]. Among them, seven diketopiperazines (**296**–**302**) contain sulfur atoms, especially a disulfide bridge in **296**–**298** and a trisulfide bridge in **299** and **300**. One of the two modified amino acid residues is substituted by a bromine, an iodine, and a chlorine atom in **296**, **297**, and **300**, respectively, and these halogenated moieties also feature a rarely occurring 1,2-oxazine ring. During the structure elucidation of **301** and **302**, the ^1^H and ^13^C NMR signals of thiomethyl groups were found to resonate at δ_H_ 1.67–2.45 and δ_C_ 12.7–18.0, differing greatly from those of oxygen- and nitrogen-bearing methyl groups. ECD spectra played an important role in determining absolute configurations for these sulfides. Three other diketopiperazines (**303**–**305**) possess at least one modified amino acid residue, and the symmetrical member (**304**) was assigned the absolute configuration by X-ray diffraction. Trichoderide A (**306**) represents the only cyclotetrapeptide from marine-derived *Trichoderma*, and all the amino acid residues were determined to feature *R*-configuration on the basis of acid hydrolysis followed by chiral HPLC analysis. In spite of the difference in ring sizes, both **305** and **306** have ornithine and succinic acid residues.

**Fig. 29:**
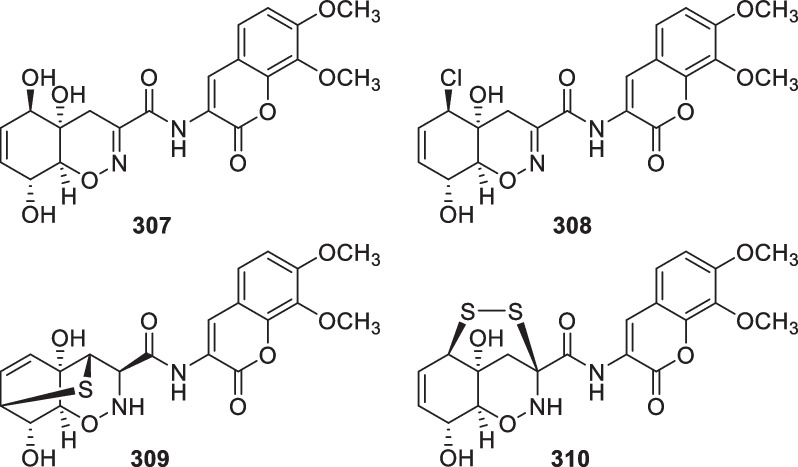
Dipeptides from marine-derived *Trichoderma*

Four linear dipeptides (**307**–**310**, Fig. 29) were obtained from *T.* *virens* and *T.* *harzianum* as well as an unidentified strain [70, 112, 113]. Despite the different sources, these isolates exhibit high structural similarities. Besides the 1,2-oxazine ring that exists in diketopiperazines **296**–**300**, a coumarin motif substituted by two methoxy groups is also present in these four metabolites. The connectivity of oxazine and α-pyrone functionalities in **307** was confirmed by X-ray diffraction analysis. The difference between **307** and **308** is the replacement of a hydroxy group in the former by a chlorine atom in the latter. The chemical total syntheses of **307** and **308** as well as their analogs were achieved by at least three groups, and their focus was the construction of the oxazadecalin core [168–172]. Trichodermamide G (**309**) and dithioaspergillazine A (**310**) also feature a sulfide bridge with one or two sulfur atoms, respectively, located in only one amino acid residue. These two sulfides were assigned absolute configurations by analysis of their ECD spectra aided by quantum chemical calculations. A series of dipeptides with the same or similar structures have also been discovered from other fungal genera, such as *Aspergillus*, *Penicillium*, and *Spicaria* [173–175].

**Fig. 30:**
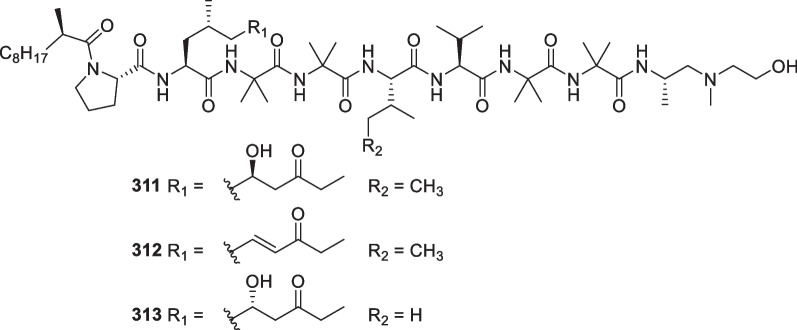
Aminolipopeptides from marine-derived *Trichoderma*

**Fig. 31:**
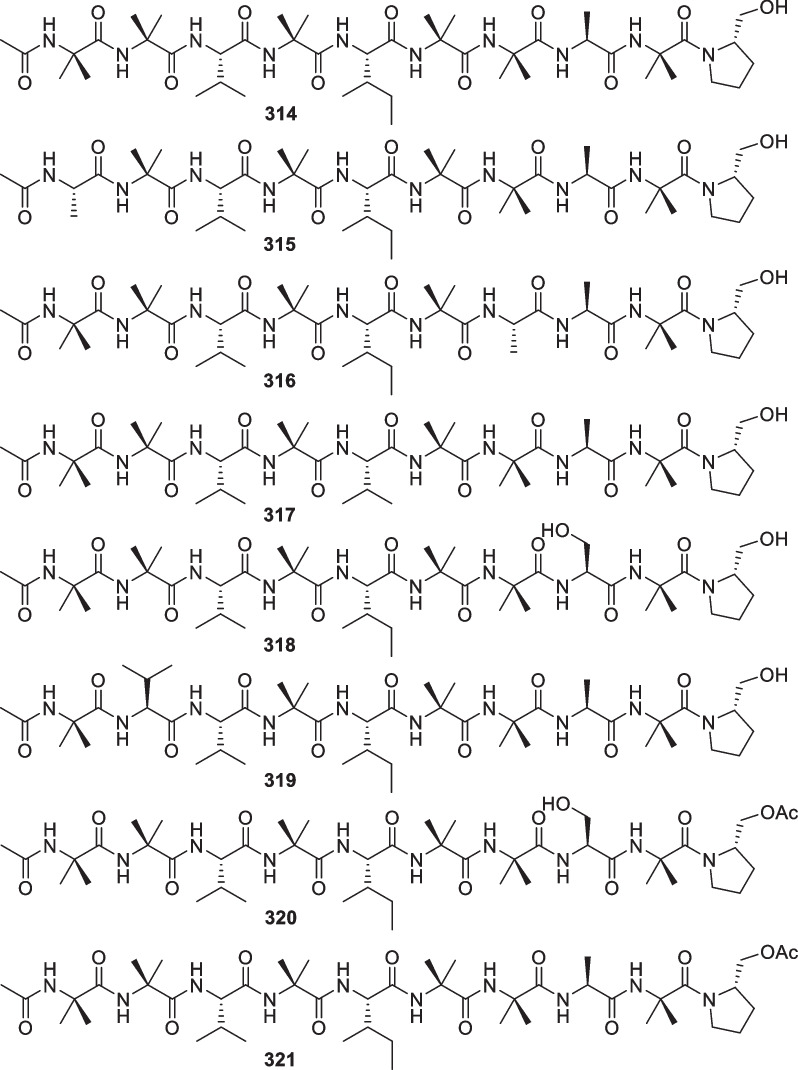
10-Residue peptaibols from marine-derived *Trichoderma*

**Fig. 32:**
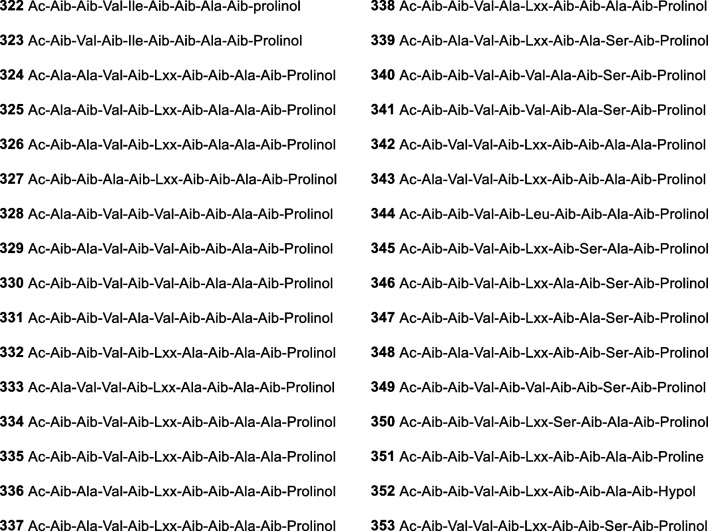
9/10-Residue peptaibols and a 10-residue peptaibiotic from marine-derived *Trichoderma* (Lxx: Leu/Ile)

**Fig. 33:**
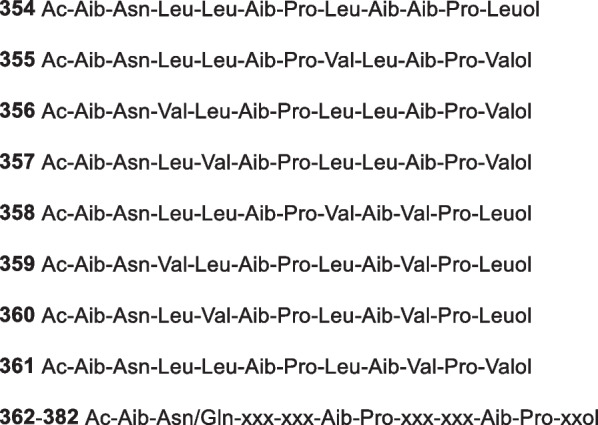
11-Residue peptaibols from marine-derived *Trichoderma* (xxx: Val/Iva/Leu/Ile, xxol: Valol/Ivaol/Leuol/Ileol)

**Fig. 34:**
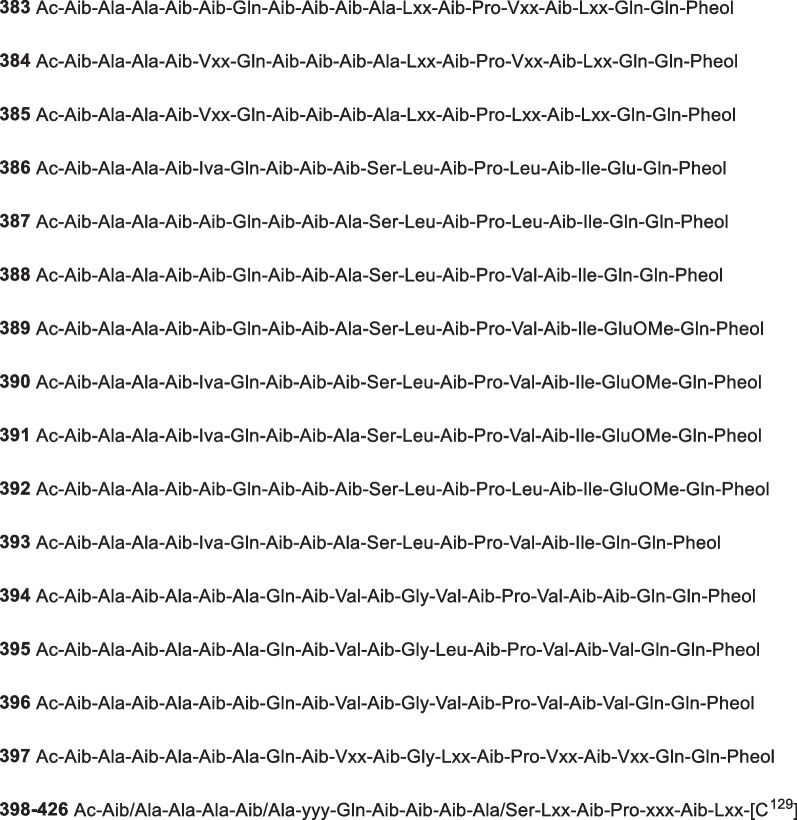
19/20-Residue peptaibols and 17-residue peptaibiotics from marine-derived *Trichoderma* (Lxx: Leu/Ile, Vxx: Val/Iva, xxx: Val/Iva/Leu/Ile, yyy: Aib/Ala/Val/Iva)

Marine-derived *Trichoderma* species have contributed a large number of linear polypeptides (**311**–**426**, Figs. 30, 31, 32, 33, 34) with 9 to 20 amino acid residues, accounting for ca. 10% of all the *Trichoderma*-derived peptaibiotics (over 1000) [176, 177]. In view of the definitions given in literature [178], all of them can be sorted into aminolipopeptides, peptaibols, and other peptaibiotics, characterized by high contents of α-aminoisobutyric acid (Aib) and acylated N-termini. In the aminolipopeptide subfamily, there are only three members (**311**–**313**), arising from an unidentified *Trichoderma* strain [114]. Besides four Aibs, the non-proteinogenic 2-methyl decanoic acid, 2-amino-6-hydroxy-4-methyl-8-oxodecanoic acid (AHMOD), and 2-[(2′-aminopropyl) methylamino] ethanol residues are present in all the three isolates. These chiral units in **311** were assigned the absolute configurations by total synthesis [115]. Besides an α-methyl-branched fatty acid, the linkage between proline and a lipoamino acid residue at N-terminus conforms to the standard of this type. In the peptaibol subfamily, a total of 83 members (**314**–**350** and **352**–**397**) with 9, 10, 11, 19, and 20 residues were identified from four marine-derived *Trichoderma* species, including *T.* *asperellum* [116–118], *T.* *longibrachiatum* [119, 120, 123], *T.* *atroviride* [121, 122], and *T.* *orientale* [124]. Each member contains a β-amino alcohol or its acetate at C-terminus, derived from the reduction of the corresponding amino acid precursor [121]. The identification of **314**–**321** and **386**–**393** was performed by analysis of the NMR and mass spectroscopic data of pure compounds, and X-ray diffraction was used to determine the structure and relative configuration of **317**. Their absolute configurations were confirmed by acid hydrolysis followed by derivation with Ru(D_4_-Por*)CO or Marfey’s reagent [116, 117, 122]. Other peptaibol isolates were mainly identified by interpretation of mass spectra given by various techniques, such as ultrahigh pressure liquid chromatography/electrospray ionization tandem mass spectrometry (UHPLC/ESI–MS/MS) [118], electrospray ionization ion-trap mass spectrometry (ESI-IT-MS) [119–121, 123, 124], collision-induced dissociation mass spectrometry (CID-MS) [120], and gas chromatography/electron impact mass spectrometry (GC/EI-MS) [119, 120, 123]. Although **351** from *T.* *asperellum* was claimed as a peptaibol in literature, it should belong to the peptaibiotic subfamily due to the lack of an amino alcohol at C-terminus [118]. Additionally, 29 unprecedented peptaibiotics (**398**–**426**) with 17 amino acid residues were identified from *T.* *atroviride* [121]. In addition to nine constant amino acid residues, a peculiar residue with a mass of 129 Da appears at C-terminus of these peptaibiotics. Unfortunately, the differentiation between some amino acid residues and their isomers, such as leucine/isoleucine, valine/isovaline, leucinol/isoleucinol, and valinol/isovalinol, remains not completed in many peptaibols and other peptaibiotics. Natural peptaibiotics have been found in some 30 known genera of fungi [121], especially in mycoparasitic ones of the Hypocreales [177].

### Alkaloids

**Fig. 35:**
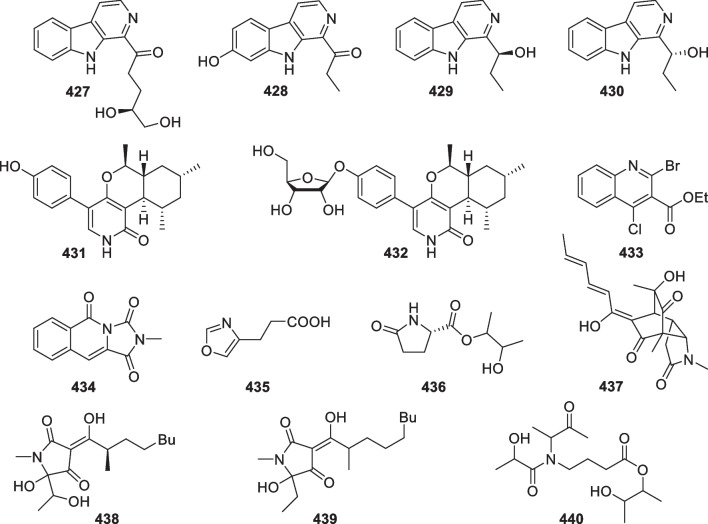
Alkaloids from marine-derived *Trichoderma*

Except for 17 nitrogen-bearing terpenes and 131 peptides, there are also 14 other nitrogenous metabolites (**427**–**440**, Fig. 35) from *T.* *asperellum* (1 compound) [25], *T.* *atroviride* (2) [105, 110], *T.* *citrinoviride* (2) [128], *T.* *harzianum* (1) [98], *T.* *reesei* (1) [81], *T.* *virens* (1) [127], and five unidentified strains (6) [125, 126]. An unidentified *Trichoderma* strain of deep sea origin (-3300 m) afforded β-carboline alkaloids **427**–**430**, with the latter two being synthesized previously. The absolute configurations of **427**, **429**, and **430** were determined by comparison of experimental and calculated specific optical rotation data. Trichodins A (**431**) and B (**432**) harbor an α-pyridone ring fused with a monoterpene unit. Regardless of the stereochemistry, these two alkaloids seem formed by reaction of deoxy-PF1140 with phenol and ribosylated phenol, respectively [179]. A quinoline motif substituted by both chlorine and bromine atoms exists in the new natural product **433**, and an imidazo[1,5-*b*]isoquinolone tricyclic system is present in **434**. As a naturally occurring compound, 4-oxazolepropanoic acid (**435**) features an oxazole ring, that has also been found in other metabolites of *Trichoderma* origin [20]. Seven members, including **436**–**442**, contain one or more pyrrole rings. The pyrrolidin-2-one unit in **437** is possibly incorporated into a sorbicillinoid precursor by a Diels–Alder reaction, and this class of molecules are not rich in nature [81]. Tetramic acids that possess a 2,4-pyrrolidinedione ring are widespread in terrestrial and marine organisms [180], but only two members (**438** and **439**) with the similar scaffold have been discovered from a marine-derived *Trichoderma* species. One C_3_ and three C_4_ units construct the acyclic **440**, which partially resembles diketopiperazine **305** of the same origin. In general, most of the heterocyclic units have been reported in other *Trichoderma* metabolites, but the halogenation in **433** and the ring system in **434** are peculiar to some extent.

### Others

**Fig. 36:**
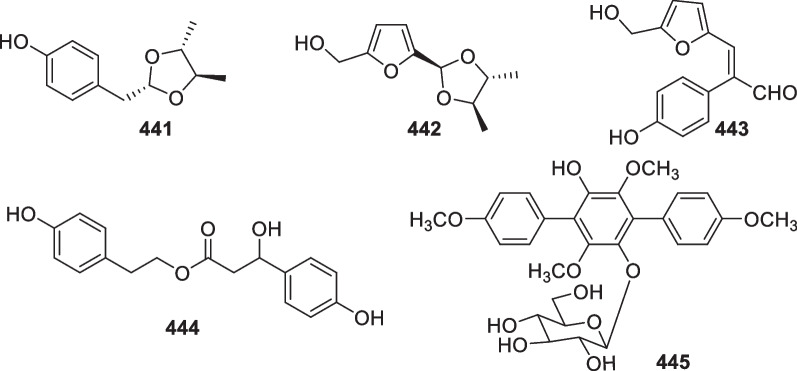
Other metabolites from marine-derived *Trichoderma*

Five other metabolites (**441**–**445**, Fig. 36), possibly arising from amino acids or sugars, were obtained from *T.* *atroviride* (1 compound) [110], *T.* *koningii* (3) [77, 129], and *T.* *reesei* (1) [130]. Among them, both **441** and **442** possess an acetal linkage due to the reaction of 2,3-butandiol with 4-hydroxyphenylacetaldehyde (4-HPAA) or 5-hydroxymethylfurfural (5-HMF), while **443** seems formed through aldol condensation between 4-HPAA and 5-HMF. 4-HPAA is an intermediate during the conversion of tyrosine to tyrosol via tyramine or 4-hydroxyphenylpyruvate [157], and 5-HMF arises from hexoses, such as glucose and fructose, via dehydration [181]. A tyrosol residue exists in **444**, of which the 3-hydroxy-3-(*p*-hydroxyphenyl)propanoate moiety also looks like a tyrosine derivative. Gliocladinin D (**445**) is a terphenyl glycoside, and its core is initially condensed between two molecules of 4-hydroxyphenylpyruvate under the catalysis of a tridomain nonribosomal peptide synthetase [182]. Terphenyls have been detected in fungi for a long time [183], but their occurrence in *Trichoderma* is really poor.

## Biological activity

### Antimicroalgal

Eight marine microalgae (phytoplankton), including *Amphidinium* *carterae*, *Chattonella* *marina*, *Heterocapsa* *circularisquama*, *Heterosigma* *akashiwo*, *Karlodinium* *veneficum*, *Prorocentrum* *donghaiense*, *Phaeocystis* *globosa*, and *Scrippsiella* *trochoidea*, that can give rise to harmful algal blooms, have been employed to evaluate the antimicroalgal (algicidal) activity of metabolites from marine-derived *Trichoderma*. As a result, 113 isolates, including 89 sesquiterpenes, 12 diterpenes, three steroids, eight polyketides, and one peptide, exhibit more or less inhibition of the phytoplankton tested (Tables 1, 2, 3). The half maximal inhibitory concentrations (IC_50_) of 109 metabolites and two ferric complexes are shown in Table 4, and the remaining four ones (**162**, **163**, **165**, and **178**) display no more than 85% inhibitory rates against *A.* *carterae*, *C.* *marina*, *H.* *akashiwo*, *P.* *donghaiense*, *P.* *globosa*, and/or *S.* *trochoidea* at a concentration of 80 or 100 μg/mL [63–65, 68]. It is worth mentioning that 19 molecules, including **15**, **32**, **65**, **68**, **69**, **93**–**95**, **104**, **106**, **107**, **114**, **115**, **129**, **149**, **167**, and **278**–**280**, that account for 16.8% of all the active ones, possess excellent inhibition effects on one or more microalga species, with IC_50_ values being ≤ 1.0 μg/mL. The former 15 members are involved in five types of terpenes, inclusive of cyclonerane, bisabolane, carotane, cadinane, and harziane derivatives.

**Table 4:** Antimicroalgal activity of 109 metabolites from the marine-derived *Trichoderma*

Compound	IC_50_ (μg/mL)						References
	*Amphidinium* *carterae*	*Chattonella* *marina*	*Heterocapsa* *circularisquama*	*Heterosigma* *akashiwo*	*Karlodinium* *veneficum*	*Prorocentrum* *donghaiense*	
**6**		12		13	3.8	20	[27]
**7**		5.2		8.0	10	9.9	[28]
**8**		8.8		21	76	6.5	[28]
**9**		61		73	71	40	[28]
**10**		2.4		26	3.9	20	[28]
**11**		5.8		37	5.5	15	[28]
**12**		59		14	35	7.3	[28]
**15**		0.66		23	2.2	37	[30]
**16**		9.9		75	14	66	[30]
**17**		12		68	41	55	[30]
**18**	na^a^	55				35	[31]
**19**	na^a^	24				na^a^	[31]
**22**				17	8.1	51	[33]
**23**				70	22	54	[33]
**24**	4.2	2.5		3.3		5.1	[34]
**25**	4.5	5.3		3.1		5.2	[34]
**26**		13		73	6.3	34	[28]
**27**	3.1	3.1		5.4		5.6	[34]
**28**		2.1		46	15	54	[27]
**Fe(28)**_**3**_		0.12		2.7	0.21	0.99	[27]
**29**		5.1		55	19	40	[27]
**Fe(29)**_**3**_		0.68		10	2.1	2.1	[27]
**30**		2.6		39	51	9.6	[27]
**31**		10		6.9	31	7.0	[27]
**32**		2.1		0.63	49	34	[27]
**33**		1.1		10	23	49	[27]
**34**		30		3.5	87	74	[27]
**35**		7.5		11	8.1	11	[27]
**36**		24		5.2	70	7.3	[35]
**37**		11		19	19	6.6	[35]
**39**		wa^b^		30	27	wa^b^	[25]
**40**		62		78	56	na^a^	[36]
**41**		6.9		2.2	5.2	48	[36]
**42**		50		85	53	na^a^	[36]
**43/44**	na^a^	8.5				19	[37]
**45**	1.8	12				37	[37]
**46**	1.6	7.0		4.2		3.4	[34]
**47**	5.2	1.6		13		1.7	[34]
**48**	6.6	8.4		11		5.7	[34]
**49**	2.0	4.8		2.5		6.8	[34]
**50**	4.6	6.4		7.1		4.7	[34]
**52**		15		8.4	10	14	[25]
**54**		6.7		2.9	6.6	10	[38]
**55**		5.4		5.8	8.4	14	[38]
**56**		3.7		6.9	9.4	12	[38]
**57**		3.3		6.5	4.6	20	[36]
**58**		5.7		14	4.0	2.6	[36]
**59**		18		34	26	42	[36]
**60**		16		41	12	33	[36]
**61**		2.1		3.8	1.9	2.5	[36]
**62**		11		4.6	12	23	[38]
**63**		1.2		4.3	1.3	5.7	[38]
**64**		3.3		9.2	1.5	6.8	[38]
**65**		0.93		7.8	2.7	4.9	[38]
**66**	23	15		25		24	[39]
**67**	71	11		83		33	[39]
**68**	1.4	0.54		6.2		5.7	[34]
**69**	12	3.4		1.0		4.5	[34]
**70**	4.6	5.4		7.2		8.3	[34]
**71**	4.6	2.2		5.0		3.9	[34]
**72**	3.5	3.3		6.3		8.4	[34]
**82**	97	26				35	[31]
**83**	21		13	16		19	[43]
**84**	53		66	63		wa^b^	[43]
**85**	14		31	25		wa^b^	[43]
**86**	wa^b^		28	30		23	[43]
**87**	68		wa^b^	64		55	[43]
**88**	20		18	24		wa^b^	[43]
**89**	25		22	34		wa^b^	[43]
**93**		0.24		3.1	5.2	3.8	[44]
**94**		0.33		4.4	7.6	3.2	[44]
**95**		0.27		4.2	5.0	3.6	[44]
**98**		1.2		6.2	12	6.5	[44]
**99**	24		19	22		10	[45]
**100**	4.7		9.8	9.2		8.3	[45]
**102**		1.8		1.1	2.1	8.9	[46]
**103**		4.3		2.7	3.2	5.8	[46]
**104**	1.4	0.54		5.4		4.3	[34]
**105**	6.7		2.7	1.5		2.4	[45]
**106**	2.4		1.8	0.88		0.68	[45]
**107**	1.8		5.2	2.1		0.54	[45]
**108**	8.6		11	1.1		3.2	[45]
**112**	6.2		3.6	3.2		1.7	[45]
**113**	1.7		1.3	2.3		1.4	[45]
**114**	1.5		2.6	0.87		0.83	[45]
**115**	2.1		1.6	0.96		0.22	[45]
**117**		2.8		56	54	54	[30]
**118**	na^a^	13				26	[37]
**122/123**	55	1.2				na^a^	[37]
**129**		2.6		2.2	0.58	1.2	[54]
**130**		6.9		3.1	4.5	7.7	[46]
**145**		7.0		42	24	70	[30]
**146**	34	31		35		18	[59]
**149**		1.9		1.0	2.5	1.4	[54]
**152**	32	25		22		18	[59]
**153**	47	22		31		20	[59]
**154**		1.2		1.3	3.2	4.3	[30]
**155**	33	26		35		29	[59]
**166**	na^a^	4.2				8.7	[37]
**167**		0.56		0.37	0.59	0.27	[66]
**168**	44	14		71		19	[39]
**177**	71	29		35		7.4	[39]
**186**		4.2		7.2	8.5	6.9	[25]
**187**		30		35	39	37	[25]
**278**		0.41		na^a^	1.0	na^a^	[103]
**279**		0.56		na^a^	0.87	na^a^	[103]
**280**		0.41		na^a^	0.69	na^a^	[103]
**289**		4.4		9.1	20	5.9	[33]
**304**		12/47		70/276	83/327	89/351	[35]

^a^na = no activity at 100 or 128 μg/mL

^b^wa = weak activity at 100 or 128 μg/mL

Structure–activity relationship analyses of cyclonerane sesquiterpenes reveal that the side chain and cyclopentane ring of **15** (IC_50_ 0.66 μg/mL) play important roles in antagonism against *C.* *marina*. Although the activity of **33** (IC_50_ 1.1 μg/mL) against *C.* *marina* also guarantees this deduction, the nitrogenous unit at the side chain terminus slightly reduces the inhibition effect. In contrast with **6** (IC_50_ 13 μg/mL), the high inhibition ability of **32** (IC_50_ 0.63 μg/mL) against *H.* *akashiwo* undoubtedly arises from the nitrogenous side chain at C-10. The enhanced inhibition against *H.* *akashiwo* due to the nitrogenous unit is also reflected by comparison of activities between **15** (IC_50_ 23 μg/mL) and **33** (IC_50_ 10 μg/mL). On the other hand, hydroxamic acids **28** and **29** do not feature attractive antimicroalgal activity, but their ferric complexes, Fe(**28**)_3_ and Fe(**29**)_3_, possess really more inhibition than themselves [27]. It is interesting that these two ferric complexes have no lethality to the marine zooplankton *Artemia* *salina* that has often been employed for screeing toxins. In view of the high and broad-spectrum activity and low toxicity of Fe(**28**)_3_, it is promising to be applied as a potent inhibitor to control harmful algal blooms in mariculture.

Among bisabolanes and norbisabolanes, only **65**, **68**, and **69** exhibit IC_50_ values being ≤ 1.0 μg/mL in the antagonistic assay against microalgae. Their high effects may correlate with the presence of a pyran unit, but it is not the only reason by consideration of the activities of its analogs, such as **54**–**56** and **62**–**70**. Stereochemistry of the pyran unit and substitution of the side chain and cyclohexane ring also influence the antagonism. Compounds **65** and **68** have the same relative configurations at C-9 and C-11, which may contribute to their high activities against *C.* *marina*. As in **69**, the variation of relative configurations at C-9 and C-11 decreases the effect on *C.* *marina*, but increases the ability to inhibit *H.* *akashiwo*. Unfortunately, the aminoglycoside unit in **54**–**56** fails to effectively improve the antimicroalgal activity.

Carotane sesquiterpenes **93**–**95** have growth inhibition against *C.* *marina*, with IC_50_ values ranging from 0.24 to 0.33 µg/mL. The carbonyl group at C-3, hydroxy group at C-11, and epoxy group at the seven-membered ring have been indicated to be the key functional groups [44]. Of the 16 cadinanes, five members (**104**, **106**, **107**, **114**, and **115**) possess inhibition against *C.* *marina*, *H.* *akashiwo*, and/or *P.* *donghaiense*, with IC_50_ values of 0.22–0.96 μg/mL. Similar to **95**, both **104** and **106** feature an isopropanol unit that likely contributes to the high activities of these two molecules. On the other hand, the methyl ester group attached to a double bond or an aromatic ring may enhance the ability to inhibit *H.* *akashiwo* and *P.* *donghaiense* by consideration of the high activities of **106**, **107**, **114**, and **115**. As for the diterpenes, harzianes **129** and **149** are more inhibitory against *K.* *veneficum* and *H.* *akashiwo*, respectively, than the others tested. The double bond at C-9 seems helpful for their inhibition, but the hydroxy group at C-3 has obvious negative effect.

Among three ergosterol derivatives, the highly transformed steroid **167** features high inhibition against *C.* *marina*, *H.* *akashiwo*, *K.* *veneficum*, and *P.* *donghaiense*, with IC_50_ values ranging from 0.27–0.59 μg/mL [66]. Despite the significant activities of this molecule against all the microalgae tested, its structure–activity relationship fails to be speculated due to the lack of any analog. The broad-spectrum inhibition ability of this steroid derivative suggests its application potential in suppressing algal blooms, which will depend on more research on mechanism exploration and toxicity evaluation. Relative to this broad-spectrum algicide, polyketides **278**–**280** have inhibition effects on only two, *C.* *marina* and *K.* *veneficum*, of the four microalgae tested [103]. Their slight differences in activity suggest the configurations of two chiral carbons that link a hydroxy group and a methoxy group, respectively, to be weak to influence the algicidal ability.

Antimicroalgal mechanisms of **68** and **104** against *C.* *marina* have been preliminarily demonstrated by detecting superoxide dismutase (SOD) and malondialdehyde (MDA) as well as soluble protein (SP) under the influence of these two metabolites, respectively [34]. Both **68** and **104** can effectively decrease the SOD activity and increase MDA and SP levels of *C.* *marina*, intimating the damage to antioxidant and membrane systems.

### Zooplankton-toxic

Besides phytoplankton, zooplankton are also widespread in the marine ecosystem and play indispensable roles in the global fishery. A low number of metabolites, **3**–**5**, **38**, **53**, **57**, **61**, **93**, **98**, **101**, **128**, **129**, **143**, **149**, **162**, and **165**, have been reported to be toxic to the zooplankton *Artemia* *salina*, a brine shrimp species. All these compounds belong to the terpene family, and their toxicities seem not very strong. As metabolites of *T.* *asperellum*, **3**–**5**, **38**, **53**, **128**, and **143** have inhibitory rates of 52.2–78.7% at 100 μg/mL, while **57** and **61** possess half maximal lethal concentration (LC_50_) values of 48 and 62 μg/mL, respectively [26, 36]. As metabolites of *T.* *virens*, sesquiterpenes **93**, **98**, and **101** are toxic to this zooplankton with LC_50_ values of 66, 56, and 21 μg/mL, repectively [44]. As metabolites of *T.* *longibrachiatum*, diterpenes **129** and **149** have LC_50_ values of 23.1 and 19 μg/mL, respectively [53, 54]. In addition, **162** from *T.* *citrinoviride* and **165** from *T.* *harzianum* also possess lethal effects on *A.* *salina*, with LC_50_ values of 65.6 and 112 μg/mL, respectively [63, 65]. Apart from these hypotoxic molecules, there are also many nontoxic metabolites in the above or other *Trichoderma* species of marine origin [27, 44, 103]. In a whole, toxicities to zooplankton are not comparable to antimicroalgal activities for the metabolites from marine-derived *Trichoderma*.

### Antibacterial

Regardless of unidentified strains, more than 20 Gram-positive and Gram-negative bacterial species, such as *Acinetobacter* *baumannii*, *Aeromonas* *hydrophilia*, *Bacillus* *subtilis*, *Enterococcus* *faecalis*, *E.* *faecium*, *Escherichia* *coli*, *Helicobacter* *pylori*, *Klebsiella* *pneumonia*, *Mycobacterium* *smegmatis*, *M.* *bovis*, *M.* *tuberculosis*, *Photobacterium* *angustum*, *Pseudoalteromonas* *citrea*, *Pseudomonas* *aeruginosa*, *Staphylococcus* *aureus*, *S.* *epidermidis*, *Vibrio* *anguillarum*, *V.* *harveyi*, *V.* *parahaemolyticus*, *V.* *splendidus*, methicillin-resistant *S.* *aureus* (MRSA), vancomycin-resistant *E.* *faecalis* (VRE), and vancomycin-resistant *E.* *faecium*, were used for antibacterial evaluation. Most of them are pathogens for human beings and aquacultural organisms. A total of 78 metabolites, comprising 47 terpenes, three steroids, 11 polyketides, 14 peptides, one alkaloid, and two others, feature bacteriostatic activities (Tables 1, 2, 3). However, most of these compounds have only moderate to weak inhibition of one or more bacterial species.

If a 10 μg/mL minimum inhibitory concentration (MIC) is taken as the threshold [184], only nine metabolites are regarded as active agents. Sesquiterpene **119** suppresses the aquatic pathogen *V.* *harveyi* with an MIC value of 4 μg/mL, while sorbicillinoids **213** and **214** inhibit *A.* *hydrophilia* at the same level [49]. Sorbicillinoid derivative **231** has been detected to inhibit *S.* *aureus*, VRE, *P.* *aeruginosa*, and *K.* *pneumonia*, with MIC values of 3.32, 1.63, 6.65, and 6.65 μg/mL, respectively [84]. During the same detection, **295** possesses an MIC value of 12.9 μg/mL to suppress *B.* *subtilis* and VRE, suggesting the γ-lactone moiety of **231** to be the key pharmacophore. Chromones **290** and **291** inhibit four *H.* *pylori* strains with MIC values of 2–8 μg/mL, and the former also suppresses four *S.* *aureus* strains, including MRSA, and one *E.* *faecalis* strain as well as one *E.* *faecium* strain within the same MIC range [94]. However, these two chromone derivatives have no activity against the Gram-negative *E.* *coli* and *P.* *aeruginosa* at 64 μg/mL. Aminolipopeptides **311**–**313** have MIC values of 0.02–2.0 μg/mL against *M.* *smegmatis*, *M.* *bovis*, and *M.* *tuberculosis* under both aerobic and hypoxic conditions, and the AHMOD moiety in **311** and **313** has been deduced to play a key role in anti-mycobacterial activity [114].

Peptaibols **386**–**393** have stronger inhibition of environmental bacteria than of laboratory ones, though the effects are not much attractive [122]. As a gram-negative bacterium, *E.* *coli* seems more resistant to these peptaibols than the gram-positive ones [122]. Compounds **162** and **442** also comply with this regular pattern in antagonism to *E.* *coli* and *S.* *aureus*, but **163** inhibits *E.* *coli* rather than *S.* *aureus* [63, 64, 77]. During the disk diffusion assays, 43 compounds, including **5**, **15**–**19**, **22**–**24**, **27**, **38**, **39**, **49**, **52**–**56**, **62**–**70**, **72**, **82**, **102**, **103**, **117**, **129**, **143**, **145**, **154**, **167**, **168**, **177**, **178**, **186**, **187**, and **289**, can inhibit the gram-negative *Vibrio* species with inhibitory zone diameters of 6–11 mm at a concentration of 20, 40, 50, or 100 μg/disk. As an outstanding representative, **177** has also been assayed for antagonistic activity using the double dilution method, but it features only MIC values of 13–50 μg/mL against the four *Vibrio* species tested [39]. Less than the number of *Vibrio* antagonists, 23 metabolites, inclusive of **24**, **27**, **46**–**49**, **63**–**66**, **68**–**71**, **102**–**104**, **129**, **166**–**168**, **177**, and **289**, can suppress the gram-negative *P.* *citrea*. Of these, **289** is the most effective one as seen from the disk diffusion test, but it feature only an MIC value of 16 μg/mL in the microdilution detection [33].

### Antifungal

The production of fungicidal antibiotics is one of the key mechanisms for *Trichoderma* to control phytopathogenic fungi [18]. However, only 25 new metabolites, **77**–**81**, **83**–**85**, **88**, **89**, **116**, **119**, **125**, **167**, **180**, **191**, **213**, **214**, **220**, **250**, **290**, **308**, **395**, **397**, and **431**, from marine-derived *Trichoderma* have antifungal activity against plant or human pathogens (Tables 1, 2, 3). Trichothecane sesquiterpenes, with active members accounting for over a half, are protruded in antagonism to yeast-like and filamentous fungi. Of them, **77**–**81** possess MIC values ranging from 1.6 to 50 μg/mL against *Candida* *albicans* and *Cryptococcus* *neoformans*, with **77** being the most potent (MIC values 3.1 and 1.6 μg/mL, respectively) [41, 42]. Structure–activity relationship analyses reveal that substituents at C-4 and C-16 greatly influence the fungistatic potency. Additionally, **83**–**85**, **88**, and **89** have MIC values of 32 or 64 μg/mL to inhibit *Botrytis* *cinerea*, *Cochliobolus* *miyabeanus*, *Fusarium* *oxysporum* f. sp. *cucumerium*, *F.* *oxysporum* f. sp. *niveum*, or *Phomopsis* *asparagi* [43]. The low MIC values (4.0–16 μg/mL) of trichodermin suggest that deacetylation and glycosylation at C-4 and hydroxylation and acetoxylation at C-16 are detrimental to the antifungal ability.

If a threshold as in antibacterial evaluation is used, most of the other plant-pathogenic antagonists seem not much attractive. Eleven pathogens of the genera *Alternaria*, *Ceratobasidium*, *Colletotrichum*, *Curvularia*, *Fusarium*, *Penicillium*, and *Physalospora* have been employed to detect the effects of **119**, **213**, and **214**, but only *Curvularia* *spicifera* appears sensitive to the former two metabolites with MIC values of 8 μg/mL [49]. **116** suppresses the pathogenic fungi *Colletotrichum* *gloeosporioides* and *F.* *oxysporum* with MIC values of 50 and 100 μg/mL, respectively, the same as those of triadimefon [48]. **125** inhibits *Colletotrichum* *lagrnarium* and *C.* *fragariae* with MIC values of 8 and 16 μg/mL, respectively, but it is relatively weak to antagonize *B.* *cinerea* from grape (64 μg/mL) and strawberry (32 μg/mL), *F.* *oxysporum* f. sp. *cucumerinum* (128 μg/mL), and *F.* *oxysporum* f. sp. *lycopersici* (256 μg/mL) [51]. **167** possesses inhibition of *Glomerella* *cingulata* with an MIC value of 12 μg/mL [66]. **180** has been assayed for inhibitory activity against *B.* *cinerea*, *Pestallozzia* *theae*, *Phytophthora* *parasitica*, *Magnaporthe* *oryzae*, and *Valsa* *mali*, with the latter two species being the most sensitive to this metabolite (MICs 8.63 and 34.5 μM) [70]. **191** and **220** have an MIC value of 125 μg/disk and a median effective dose (ED_50_) of 9.13 μg/mL, respectively, to inhibit *Pestalotiopsis* *theae* through the paper disk dilution method, rather than the conventional broth dilution test [76, 82].

Besides trichothecanes, several other metabolites are also antagonistic to human pathogens. Chromone **290** has an MIC value of 16 μg/mL against two *C.* *albicans* strains and an MIC value of 64 μg/mL against one *Aspergillus* *fumigatus* strain [94]. Dipeptide **308** inhibits amphoterocin-resistant *C.* *albicans* with an MIC value of ca. 15 μg/mL, with the chlorine atom being a pharmacophore [112]. Among polypeptides, peptaibol **395** possesses only 63% inhibitory rate against *A.* *fumigatus*, a human-pathogenic fungus, at 100 μg/mL [123], while **397** inhibits *C.* *albicans* ATCC 10231 from bronchomycosis, 247 FN from finger nails, 311 FN from finger nails, and 098 VC from vaginal cavity with MIC values of 2.49, 4.92, 19.66, and 2.49 μM [124]. Alkaloid **431** suppresses human-pathogenic *C.* *albicans* with an IC_50_ value of 25.38 μM, and ribosylation of the phenolic hydroxy group dramatically reduces the effect as indicated by the low activity of **432**. Although **250** possesses no antagonistic effect on *C.* *albicans*, it features synergistic antifungal activity with ketoconazole [91]. It must be said that these tests are valuable tries to use *Trichoderma* metabolites to antagonize human-pathogenic fungi.

### Cytotoxic

A series of human and murine cancer cell lines, such as A-375, A375-S2, A549, A2780, Bel-7402, CNE1, CNE2, DU-145, HCT-8, HCT-15, HCT-116, Hela, HepG2, HL-60, Huh-7, Jurkat, KB, Lovo, MCF-7, MDA-MB-435 s, NCIH-460, NCI-H929, SUNE1, SW620, P388, and L1210 cells, have been used for in vitro cytostatic evaluations, and most of them have been performed through the 3-(4,5-dimethyl-2-thiazolyl)-2,5-diphenyl-2*H*-tetrazolium bromide (MTT) method. 42 metabolites, including 10 terpenes, 26 polyketides, four peptides, and two alkaloids, have been determined to possess more or less cytotoxicities (Tables 1, 2, 3). If an IC_50_ value of 10 μM is taken as the potency threshold [184], only 12 metabolites have potent activities.

Among these potent molecules, cadinane sesquiterpene **109** possesses cytotoxicities against NCI-H929 myeloma and SW620 colorectal cancer cells with IC_50_ values of ca. 6.8 and 9.3 μM, respectively, while **110** inhibits NCIH-460 lung and SW620 colorectal cancer cell lines with IC_50_ values of ca. 7.8 and 8.6 μM, respectively. The presence of two carboxylic acid groups in **109** and **110** has been deduced to be helpful to their activities [47]. Wickerane diterpenes **156**–**161** have been assayed for cytotoxicities against HL-60, P388, and L1210 leukemia cells, but only the carbonyl-bearing **156** with IC_50_ values of 6.8–7.9 μM is potent [61, 62]. Cyclopentenones **182**–**184** significantly inhibit P388 leukemia cells with IC_50_ values of 1.69, 6.85, and 9.03 μM, respectively [71]. Bisorbicillinoid **218** features inhibition of human A549 lung and MCF-7 breast cancer cells with IC_50_ values of 5.1 and 9.5 μM, respectively [86]. Decalin derivatives **265** and **266** suppress HL-60, P388, and L1210 leukemia cells with IC_50_ values ranging from 3.8–6.7 μM [97], four orders of magnitude lower than that of **261** [95]. Dipeptides **308** and **310** are potential inhibitors for HCT-116 and Jurkat cells, with IC_50_ values of 0.71 and 1.3 μM, respectively. The chlorine atom in **308** and the disulfide bridge in **310** play key roles in their cytotoxicities [112, 113]. Dipeptide **308** can also inhibit the proliferation of Hela cells, with an IC_50_ value of 3.1 μM. Besides differences in potency, **308** also has distinct action mechanism, accumulating cells in the S phase, from several synthetic analogs. Its mechanistic specificity is probably attributed to the chlorohydrin moiety, which may contribute to developing DNA-damaging agents. However, numerous in vivo antitumor experiments are required before evaluating the therapeutic potential of this molecule [172]. In addition, peptaibol **395** has an IC_50_ value of 2.5 μM in inhibition of KB cells [123]. These positive results further strengthen the clinical potential of *Trichoderma* metabolites.

### Anti-inflammatory

The anti-inflammatory property of some metabolites has been assayed for inhibiting the production of nitric oxide (NO) in different cell lines induced by lipopolysaccharide [40, 55, 81, 88]. The number of active metabolites amounts to 21, and all of them belong to terpene and polyketide families. Trichothecane sesquiterpenes **73**–**76** reduce the lipopolysaccharide-induced NO production in microglial BV-2 cells at 10 μM, with **75** being the most potent [40]. Harziane diterpenes **132** and **133** weakly suppress the production of NO in RAW264.7 macrophages at 100 μM [55]. Sorbicillinoids **200**, **201**, **203**–**206**, **208**, **211**, and **223**–**228** feature higher anti-inflammatory effects than indomethacin, a positive control with an IC_50_ value of 41 μM. In particular, the IC_50_ values of **200**, **203**–**206**, **223**, and **224** are lower than 10 μM. Monomeric sorbicillinoid **205** with an IC_50_ value of 0.94 μM is the most potent one, and substitution modes at the phenyl core and the side chain contribute to its high activity [81]. This molecule is promising to be developed as a therapeutic agent after a series of in vitro and in vivo determination in the future. It can be concluded that substitution and oxidation of the benzene ring and side chain have important impacts on anti-inflammatory activities of this type of molecules. In a dose-dependent manner across 3–60 μM, **236** can reduce the nitrite production stimulated by lipopolysaccharide in J774A.1 macrophages [88].

### Miscellaneous

There are 25 metabolites that possess other activities, such as antiviral, phytotoxic, insect-toxic, zebrafish-toxic, antifouling, antioxidant, enzyme-inhibitory, NF-κB-inhibitory, anti-pulmonary fibrosis, anti-Aβ fibrillization, and neuroprotective properties. Sesquiterpenes **126** and **127** have moderate activities against the hepatitis C virus, with half maximal effective concentration (EC_50_) values being ca. 24.5 and 20.4 μM, respectively [52]. Harziane diterpenes **138**–**140**, **142**, **148**, and **151** exert growth inhibition of amaranth and lettuce seedlings at 200 ppm. Although they also have an inhibitory effect on amaranth roots, all of them are avirulent to the elongation of lettuce roots [57]. Besides antimicrobial and cytotoxic activities, the acute toxicity to the larvae of *Calliphora* *vomitoria* has also been detected for **395**, with the minimal effective dose (MED) being 250 μg/mg [123]. Despite the potent inhibition of *P.* *theae* by **220**, its high effects on mortality and malformation of zebrafish at a low concentration (0.625 μM) exclude the antifungal application [82]. Decalin derivative **268** prevents the settlement of *Bugula* *neritina*, a widespread fouling organism in marine environments, larvae with an EC_50_ value of 29.8 μg/mL [98]. Six metabolites, **196**, **234**–**236**, **442**, and **444**, have been evaluated to be moderate antioxidants through the 1,1-diphenyl-2-picrylhydrazyl (DPPH) radical scavenging method [77, 78, 87, 129]. Of them, **196** is also an effective inhibitor of the Aβ fibril formation and a moderate protectant against the hydrogen peroxide-induced neuronal death [78]. Koninginins **254** and **255** have moderate inhibition of protein tyrosine phosphatase 1B (PTP1B), with IC_50_ values of 53.1 and 65.1 μM, respectively [92]. In addition, naphthopyrones **276** and **277** possess IC_50_ values of 58.7 and 18.0 μΜ, respectively, against tyrosine kinase, with the bisnaphtho-γ-pyrone nucleus being responsible for the inhibition [102]. Vertinolide **230** has an IC_50_ value of 13.83 μM to inhibit TNF-α-induced NF-κB, and no cytotoxicity against the human embryonic kidney cell line 293 has been detected at 50 μM [83]. Alkaloids **427**, **429**, and **430** have been screened for inhibition of collagen accumulation, and only **427** with a ca. 85.21% inhibitory rate at 10 μM features high potency to reduce the pulmonary fibrosis [125].

## Metabolic relevance

*Trichoderma* species have their own metabolic characteristics, reflected by the diverse cycloneranes, bisabolanes, harzianes, sorbicillinoids, and peptaibols. These metabolites may exist in a broad spectrum of species, as shown in Tables 1, 2, 3. On the other hand, it is easy to be understood that different *Trichoderma* species of marine origin also produce varied metabolites. For examples, carotanes, trichothecanes, wickeranes, and decalins have been encountered in one or a small number of species, demonstrating the interspecific differences of metabolites. Moreover, *Trichoderma* strains of the same species tend to feature discrepancies in metabolic profiles, evidenced by the discovery of new compounds from each isolate. This phenomenon can be attributed to the variation of strain sources, though technical divergence and personal tendency during isolation and identification processes also make differences to some extent.

The findings of more than 400 new compounds from marine-derived *Trichoderma* commendably explain the shaping effect of marine environments on biosynthetic routes. The impacts may be caused by genic or transcriptional variations, especially for the known species that have also been reported as terrestrial-derived ones. To date, several new or known skeletons, such as cuparane, synderane, pupukeanane, harzianoic acid, citrinovirin, fusicoccane, cleistanthane, and trichorenin, have been discovered from only marine-derived strains, but their uniqueness still requires more supports from both natural product chemistry and molecular biology. As for substitution, the aminosugar unit has exclusively been found in the metabolites, such as **38**, **53**–**56**, **90**, **119**, and **124**, of marine-derived *Trichoderma* so far. Moreover, marine-derived *Trichoderma* strains have contributed a series of halogenated metabolites, including **82**, **114**, **164**, **183**, **184**, **187**, **188**, **296**, **297**, **300**, **308**, and **433**, which exhibit higher structural diversity than those from terrestrial-derived strains [20]. Halogenation is characteristic of marine natural products, and the chlorine, bromine, and iodine atoms in these metabolites come from the seawater constituents or the added sodium halides [107, 112].

Culture substrates and conditions play important roles in the secondary metabolite production of marine-derived *Trichoderma* [185]. Algicolous *Trichoderma* sp. TPU199 was chemically investigated on the basis of several different cultivations, leading to the discovery of **77**–**81**, **296**–**300**, and **310** as well as several known terpenes, peptides, and polyketides [41, 42, 107, 108, 113]. Fermented in the freshwater medium under agitation for 7 days, this strain produced trichothecane sesquiterpenes **77**, **80**, and **81** [42]. Supplemented with 3.0% NaI, the culture broth further afforded trichothecane sesquiterpenes **78** and **79** and diketopiperazines **297**–**299**, besides **77** [41]. If only NaI was replaced by NaBr, a brominated diketopiperazine (**296**) was then yielded [107]. In the same freshwater medium, a long-term (5 weeks) static fermentation induced the production of dipeptide **310** [113]. Agitated for 7 days in the seawater medium, the culture broth gave dipeptide **308** and diketopiperazine DC1149B, a chlorinated analog of **296** and **297** [107]. When 1% dimethyl sulfoxide (DMSO) was added into the seawater, rather than freshwater, medium, the production of diketopiperazine **300** was further induced under agitation for 7 days [108].

“One strain many compounds” (OSMAC) and chemical epigenetic manipulation strategies were also used to the coral-derived *T.* *harzianum* XS-20090075 [186], which resulted in the discovery of 12 new molecules [56, 57, 98, 100]. A culture for four weeks in the rice medium led to the production of harziane diterpenes **138**–**142**, **148**, and **151** [57]. When the culture period was elongated to 45 days, decalin derivatives **267** and **268** were produced in the same medium [98]. Treated with a histone deacetylase inhibitor (sodium butyrate), two new diterpenes (**137** and **164**) including a chlorinated one (**164**) were obtained from the culture in the rice medium [56]. Changed to the Czapek’s medium, the culture afforded a alkaloid (**433**) that contains both bromine and chlorine atoms [98]. In the PDA (possibly an error of PDB) medium, a static culture for 45 days yielded hydroxyanthraquinones **271** and **272** [100].

Besides the whole substrate variation, a single nutritional factor may also alter the metabolic profile of a *Trichoderma* strain. Addition of a single amino acid, l-phenylalanine, into the culture medium of *T.* *erinaceum* F1-1 elicited the production of 18 aromatic metabolites, with **283**–**288** being new ones. Simultaneously, an excess (2 g/L) of l-phenylalanine also resulted in the morphological alteration of this fungal strain. However, it was strange that only one of the divergent molecules harbors a nitrogen atom, differing from the privileged production of alkaloids in other marine fungi. The result indicated the incapability of some *Trichoderma* strains to incorporate a single amino acid into metabolic pathways [32].

## Co-culture induction

**Fig. 37:**
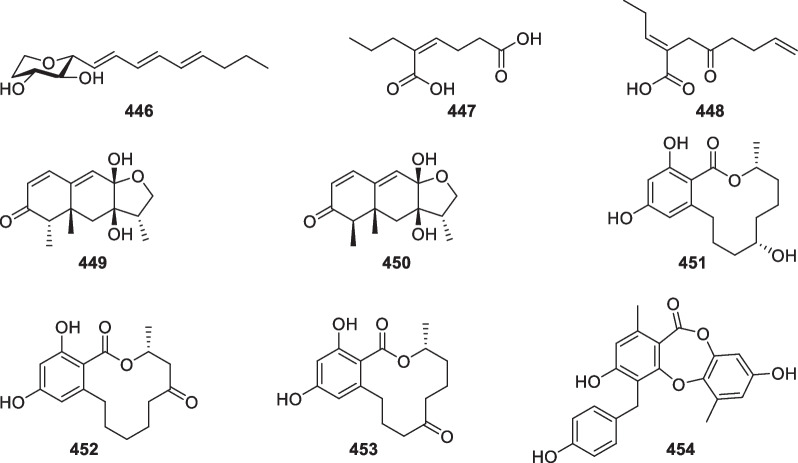
Metabolites from co-cultures of marine-derived *Trichoderma* and other microbial strains

*Trichoderma* species are always present in extremely complex microbial communities. Apart from abiotic conditions, other microbes also interfere their growth and metabolism in nature. Undoubtedly, co-culture experiments are effective approaches to simulate the actual ecological situation and awake some sleeping genes. Mixed fermentation of marine-derived *Trichoderma* strains with other fungal and bacterial strains led to the production of nine new metabolites, **446**–**454** (Fig. 37) [187–190]. Chaunopyran A (**446**) was produced at an extremely low level by monoculture of *Chaunopycnis* sp. CMB-MF028, but its productivity was greatly improved by co-cultivation with *T.* *hamatum* CMB-MF030. Both the fungal isolates were obtained from the inner tissue of the mollusc *Siphonaria* sp [187]. (*Z*)-2-Ethylhex-2-enedioic acid (**447**) and (*E*)-4-oxo-2-propylideneoct-7-enoic acid (**448**) were yielded by co-culture of *Trichoderma* sp. Gc(M2) and *Penicillium* sp. Ma(M3)V, isolated from different marine sponge species [188]. Two new sesquiterpenes (**449** and **450**) and four new polyketides (**451**–**454**) were identified from co-culture extracts of fungus *Trichoderma* sp. 307 and bacterium *Acinetobacter* *johnsonii* B2, obtained from a mangrove plant and an aquaculture pond, respectively [189, 190]. It can be concluded that *Trichoderma* species or other microbes might produce extra products due to the activation of silent metabolic routes during co-culture processes [191]. At least 75 *Trichoderma* species have the mycoparasitic ability [7], and their fermentation mixed with other fungi means more intensive competition or antagonism that may involve the large-scale production of antibiotics.

## Biotransformation and biodegradation

*Trichoderma* species exhibit biotransformation functions towards heterogeneous organic matters due to the production of enriched enzymes [192, 193], but marine-derived strains have less been examined. As the metabolites of *Chaunopycnis* sp., pyridoxatin atropisomers were transformed to a pair of methylated derivatives by the co-cultivated *T.* *hamatum* [187]. As a biogenetic precursor of many monoterpenes, geraniol was transformed to 1,7-dihydroxy-3,7-dimethyl-(*E*)-oct-2-ene by a marine-derived *Hypocrea* strain. During the process, a double bond was oxygenated to an alcohol [194]. On the other hand, three marine-derived *Trichoderma* strains effectively degraded malachite green, pentachlorophenol, and chlorpyrifos, respectively, which are industrial and agricultural agents causing environmental pollution [195–197]. These strains were obtained from mangrove, ascidian, and sponge organisms, respectively, and their degradation ability may rely on the production of exoenzymes, such as laccase and peroxidase [195, 198]. Considering the diverse substitution patterns in metabolites from marine-derived *Trichoderma*, their biotransformation and biodegradation potentials will not disappoint the relevant researchers in future.

## Conclusions

Over a duration of 30 years, marine-sourced 77 *Trichoderma* strains have contributed 445 new metabolites, including many new structural types from this genus and new carbon skeletons in nature. Their metabolic characteristics are also reflected by the high populations of irregular carbon numbers and cyclization and cleavage patterns in some scaffolds. These phenomena might arise from the oxidation by reactive oxygen species produced under saline and alkaline stresses. Halogen and sulfur atoms as well as sugar units are incorporated into 12, nine, and 15 members, respectively, especially eight aminosugar-bearing ones. The production of halides probably profits from enriched halogen anions and related enzymes. The discovery greatly adds to the molecular diversity of *Trichoderma* metabolites, especially those with cyclonerane, bisabolane, harziane, sorbicillinoid, and peptaibol frameworks. Among the metabolites, 235 members feature antimicroalgal, zooplankton-toxic, antibacterial, antifungal, cytotoxic, anti-inflammatory, and other activities, demonstrating the application potentials of marine-derived *Trichoderma* in aquaculture, agriculture, and healthcare. Marine environments probably shape the metabolic profiles of the involved *Trichoderma* strains, and culture methods seem also responsible for the metabolic diversity. Considering the mycoparasitic instinct of many *Trichoderma* species, co-culture with other fungi seems a prospective strategy to mine their valuable metabolites. On the other hand, marine-derived *Trichoderma* strains may methylate and oxidize heterogeneous molecules, and their biodegradation ability is also attractive. In view of the high structural diversity and various bioactivities, the molecules produced or transformed by marine-derived *Trichoderma* are promising candidates to develop potent drugs for human diseases and antagonistic agents in agriculture and aquaculture.

## Data Availability

All the data and materials provided in the manuscript are obtained from included references and available upon request.
